# Individual and mixture associations of placental per- and polyfluoroalkyl substance with neurodevelopment at 12 and 24 months of age

**DOI:** 10.1016/j.envadv.2025.100650

**Published:** 2025-06-14

**Authors:** Neha Sehgal, Stephanie M. Eick, Kartik Shankar, Dana Boyd Barr, Jayne B. Bellando, Ginger McCorkle, Clark R. Sims, Parinya Panuwet, Volha Yakimavets, Donald Turner, Lauren A. Havens, Andrew J. Morris, Kevin J. Pearson, Aline Andres, Todd M. Everson

**Affiliations:** a Gangarosa Department of Environmental Health, Rollins School of Public Health, Emory University, Atlanta, GA, USA; b Department of Epidemiology, Rollins School of Public Health, Emory University, Atlanta, GA, USA; c USDA Agricultural Research Service, Responsive Agricultural Food Systems Research Unit, College Station, TX, USA; d Department of Pediatrics, University of Arkansas for Medical Sciences, Little Rock, AR, USA; e Arkansas Children’s Nutrition Center, Little Rock, AR, USA; f Central Arkansas Veterans Affairs Healthcare System and University of Arkansas for Medical Sciences, Little Rock, AR, United States; g Department of Pharmacology and Nutritional Sciences, University of Kentucky, Lexington, KY, USA

**Keywords:** Neurodevelopment, Mixtures analysis, BSID-III, Placenta, PFAS

## Abstract

Maternal serum per- and polyfluoroalkyl substances (PFAS) are linked to infant neurodevelopment vulnerabilities. However, the impact of placental PFAS exposure, a more proximal *in utero* exposure estimate, remains unknown. We hypothesize that elevated placental PFAS levels are associated with delayed neurodevelopment at 12 and 24 months.

Mother-infant dyads (*n* = 151) were enrolled in a prospective cohort in Arkansas, US. PFOA, PFOS, PFHxS, PFNA, and PFDA were detected in >65 % of placentas. The Bayley Scales of Infant and Toddler Development (BSID)-III was administered at 12 and 24 months. Individual associations of each placental PFAS on BSID-III scores were examined using linear regressions. Mixture effects were assessed using quantile g-computation and Bayesian Kernel Machine Regression.

Placental PFAS were jointly associated with lower cognitive (Ψ = −2.6; 95 % CI = −6.0, 0.9) and motor (Ψ = −2.9; 95 % CI = −6.6, 0.9) scores at 12 months and higher social-emotional scores (Ψ = 3.3; 95 % CI = −1.9, 8.5) at 24 months among males. Among females, higher PFAS mixture levels were associated with higher language (Ψ = 3.3; 95 % CI = −1.2, 7.7) and social-emotional (Ψ = 3.7; 95 % CI = −3.3, 10.6) scores at 24 months. Linear regressions showed PFHxS and PFOS were associated with lower cognitive scores at 12 months and PFDA was associated with lower motor scores at 12 months among males, while PFOS and PFNA were associated with higher social-emotional scores at 24 months among females, although confidence intervals included the null. In a matched subset with maternal serum, cord serum and placental PFAS (*n* = 98), we observed mostly null associations consistently across biomatrices and noted few associations, such as a negative relation of cord PFOA and PFHxS with language scores at 12 months among females.

Overall, placental PFAS levels were modestly associated with performance on neurodevelopmental assessments in early childhood and these relationships were sex- and compound-specific, and most confidence intervals cross the null.

## 1. Introduction

Per- and polyfluoroalkyl substances (PFAS), commonly known as “forever chemicals”, are man-made chemicals ubiquitously present on non-stick cookware, stain-resistance carpets, and in numerous cosmetics (Rogers et al., 2021; Guidance on PFAS Exposure 2022; Buck et al., 2011). PFAS are persistent organic pollutants with relatively long half-lives of several years (Blake and Fenton, 2020; Panieri et al., 2022). In a nationally representative sample of the US population (NHANES years 1999–2008), numerous PFAS compounds have been detected in serum almost universally (>90 % of the entire sample population) (Kato et al., 2011). Such prevalent exposures to PFAS are a pervasive public health concern for the general US population and of paramount concern for vulnerable populations such as children. PFAS in maternal circulation can cross the placenta and reach the fetal environment (Guidance on PFAS Exposure 2021). Consequentially, children can be exposed to PFAS prenatally via transplacental transfer to cord blood. Furthermore, they can have continued exposure postnatally, for example, via human milk, water, and diet (Guidance on PFAS Exposure 2021). *In utero* PFAS exposure has been linked to adverse pregnancy outcomes such as pre-term births and fetal growth restriction (Guidance on PFAS Exposure 2021), impaired growth (Guidance on PFAS Exposure 2021; Eick et al., 2021; Taibl et al., 2023; Eick et al., 2023), and delayed neurodevelopment (Guidance on PFAS Exposure 2021; Eick et al., 2021; Kim et al., 2023).

A growing body of epidemiological evidence suggests that maternal serum PFAS levels are inconsistently associated with diverse neurodevelopmental phenotypes. For example, in a birth cohort from Canada, increased maternal serum levels of legacy PFAS mixture [perfluorooctanoic acid (PFOA), perfluorooctane sulfonate (PFOS), and perfluorohexanesulfonic acid (PFHxS)] was associated with poorer performance intelligence quotient in males but had no effects on executive functioning among females and males (Goodman et al., 2023). Results from a Danish birth cohort found that increased serum levels of PFOS, PFOA, and PFNA were individually associated with increased use of grammar and syntactic complex sentences by 18- to 36-month-old children, albeit the relation was not statistically significant (Beck et al., 2023). Among mothers and children from Shanghai, China, a simultaneous 25 % increase in 10 PFAS compounds was associated with lower cognition and language scores, but higher adaptive behavior scores (Luo et al., 2022). Furthermore, prior research, including meta-analyses, has indicated prenatal PFAS to be a possible risk factor for diverse neurotypes such as autism spectrum (Oh et al., 2021; Oh et al., 2022; Shin et al., 2020; Yao et al., 2023) and attention-deficit hyperactivity phenotypes (Yao et al., 2023; Skogheim et al., 2021; Forns et al., 2020). However more recent systematic reviews (Stübner et al., 2023; Xin et al., 2023) note discrepant evidence, which could be due to varying effects from different PFAS compounds and due to heterogeneity of outcome assessments and imperfections in assessments.

One major gap in our understanding of whether PFAS compounds impact postnatal neurodevelopment trajectories lies in the exposure scenarios that are studied. Past studies have mostly examined whether maternal serum levels of PFAS prenatally influence infant neurodevelopment postnatally. PFAS levels measured in maternal serum are reflective of PFAS circulating in the mother’s blood but only partly reflect prenatal fetal exposure. Alternatively, as a marker of prenatal fetal PFAS exposure, other studies have measured PFAS in cord blood (Wu et al., 2023). Both maternal and cord blood PFAS measures may be influenced by metabolic changes during pregnancy (e.g., blood volume changes and glomerular filtration rates) that further influence the partitioning of PFAS across maternal and cord blood. The transfer of PFAS from maternal to cord blood is via the placenta. The placenta is an essential organ that forms the maternal-fetal compartment, regulates the transfer of endogenous and exogenous compounds, and to a certain extent, limits fetal exposure (Gude et al., 2004). Studies have detected PFAS in the placenta and observed increasing placental PFAS-to-maternal serum PFAS ratios with increasing gestational time across pregnancy indicating PFAS may accumulate in the placenta (Mamsen et al., 2019; Bangma et al., 2020). PFAS has been also quantified in the placenta, an organ in closer contact with the fetal environment than maternal blood and which directly modulates the *in utero* environment (Guidance on PFAS Exposure 2021). *In vitro* and *in vivo* studies have suggested the placenta as a target for PFAS (Blake and Fenton, 2020) and epidemiological studies have highlighted its association with placental molecular dysfunctions (Guidance on PFAS Exposure 2021; Liang et al., 2024; Chowdhury et al., 2024; Szilagyi et al., 2020), responses that may have adverse consequences on pregnancy and relatedly on neurodevelopment (Freedman et al., 2022; Rosenfeld, 2021). While studies have focused on maternal or cord blood associations with neurodevelopment, to our knowledge, there are no epidemiological studies that have investigated how PFAS levels in the placenta are associated with neurodevelopment during the first two years of life. Given the important roles of the placenta in establishing and promoting early growth development, including neurodevelopment, we aimed to characterize whether placental PFAS concentrations were associated with early childhood neurodevelopment.

In this analysis, we leverage a prospective birth cohort study in central Arkansas, US, to ascertain associations between placental PFAS mixtures and neurodevelopment, as measured by the Bayley Scales of Infant and Toddler Development III (BSID-III), (Bayley, 2011; Nakazawa et al., 2022) at 12 and 24 months of age. We hypothesize that higher placental PFAS levels are associated with lower cognitive, language, motor, social-emotional, and adaptive skills at both time points, indicating delayed neurodevelopment at 12 and 24 months.

## 2. Methods

### 2.1. Study population

The Glowing cohort (clinicaltrails.gov #NCT03281850) is a prospective birth cohort study that recruited 300 mother-infant dyads within the first 10 weeks of gestation from central Arkansas, and surrounding areas between 2010 and 2014. This cohort aimed to study the maternal programming of infant metabolism, growth, and development in the first 2 years of life. The study protocol and recruitment information have been detailed elsewhere (Heard-Lipsmeyer et al., 2020; Diaz et al., 2024). Mothers were eligible to participate in the study if they were: (1) second parity women with singleton pregnancies; (2) at least 21 years old; (3) body mass index (BMI) between 18.5 to 24.9 kg/m^2^ (healthy weight), or 25 to 35 kg/m^2^ (overweight or obesity); and (4) non-smokers reporting no alcohol use during pregnancy. Categorical BMI was an enrollment characteristic due to the primary study’s focus on maternal programming of child’s metabolism. Mothers with pregnancy or birth complications (e.g., preeclampsia, pre-term birth, gestational hypertension, gestational diabetes), conceptions aided with fertility treatment, pre-existing medical conditions, or sexually transmitted infections were excluded from the study. Infants with serious medical conditions were also excluded from the cohort. The Glowing cohort was approved by the University of Arkansas for Medical Sciences Institutional Review Board. All participants provided their written and informed consent prior to participating in the study.

### 2.2. Quantification of PFAS concentrations

Placentae were sampled within 30 minutes of delivery and sample processing and storage were performed within two hours. Placental samples with severe pathologies were excluded. Details of quantitation of PFAS in placenta are described elsewhere (Everson et al., 2025). Briefly, one square inch of placental tissue was collected from six random sites of the villous core, maternal and fetal sides. Samples were washed three times in ice-cold phosphate buffered saline to remove maternal blood and flash-frozen until further analysis. All six samples collected from a single placenta were pooled, frozen in liquid nitrogen, and pulverized to homogenize and ensure a comprehensive representation of placental exposure. Before quantitation at Emory University, samples were randomized to reduce potential batch effects. Powdered placental tissue samples (0.2 mg to 0.7 mg per sample) were spiked with isotopically labeled analogues of target PFAS; homogenized in methanol; extracted on a Strata RP on-line extraction column followed by chromatographic separation on a Betasil C18 analytical column, and then analyzed. Analyte concentrations were calculated using isotope dilution calibration. For quality control and assurance, PFAS levels in the samples were analyzed with serum NIST samples (although the matrix differed) alongside three blank samples and two quality control samples at two different levels to ensure no analytic contamination. Mean relative recoveries in samples spiked at 0.5 ng/mL and 5 ng/mL were 99.1 % (range 85 to 116) and 97.6 % (range 82 to 110). NIST Standard Reference Material values were within ±20 % of their certified values (Table S1).

In total, 151 placental samples were available for PFAS quantification. Using high performance liquid chromatography tandem mass spectrometry (HPLC-MS/MS), levels of 17 PFAS [namely, perfluorohexanoic acid (PFHxA), perfluorohexane sulfonic acid (PFHxS), perfluoroheptanoic acid (PFHpA), linear perfluorooctanoic acid (PFOA), summed perfluorooctane sulfonic acid (sPFOS), perfluorooctane sulfonamide (PFOSA), n-methylperfluoro-1-octanesulfonamidoacetic acid (MePFOSAA), perfluorononanoic acid (PFNA), perfluorodecanoic acid (PFDA), perfluorodecane sulfonic acid (PFDS), perfluoroundecanoic acid (PFUnDA), perfluorododecanoic acid (PFDoDA), perfluoropentanoic acid (PFPeA), n-ethylperfluoro-1-octanesulfonamidoacetic acid (EtPFOSAA), hexafluoropropylene oxide dimer acid (HFPODA; Gen X), perfluoroheptane sulfonic acid (PFHpS), and perfluorobutanesulfonic acid (PFBS)] were quantified.

In this analysis, we focused only on 5 of the 17 measured PFAS as they were detectable in >65 % of the samples (namely, PFOA, PFHxS, PFNA, PFDA, and PFOS). Samples with measured PFAS levels below the limit of detection (LOD) were imputed with LOD/√2. The LOD was 0.029 ng/g for PFOS; 0.010 ng/g for PFNA, PFHxS, and PFDA; and 0.017 ng/g for PFOA. Additionally, we calculated summed PFAS concentration levels as the sum of PFOA, PFHxS, PFNA, PFDA, and PFOS to examine the association with overall PFAS burden.

We additionally quantified PFAS in maternal serum and cord serum samples. Detailed descriptions of these methods are published elsewhere (Sims et al., 2025). Briefly, 17 PFAS were quantified via Ultra-High-Performance Liquid Chromatography coupled electrospray ionization tandem mass spectrometry. Calibrators, controls, and mass-labeled internal standards, where available, were purchased from Wellington Laboratories (Guelph, Ontario, Canada). The LOD for maternal and cord serum PFAS was 0.1 ng/mL, and concentrations below the LOD were imputed with LOD/√2. For this study, we focus on the 5 PFAS that were well-detected in placental tissues (PFOS, PFOA, PFHxS, PFNA, and PFDA), and include descriptive statistics and secondary analyses with maternal and cord serum PFAS to contextualize similarities and differences across biomatrices.

### 2.3. Neurodevelopmental assessments at 12 and 24 months

The Bayley Scales of Infant and Toddler Development-III (BSID-III) is an individually administered test that assesses the developmental functioning of young children between the ages of 1 month and 42 months (Albers et al., 2006). The test is comprised of five domains: adaptive, cognitive, language, motor, and social-emotional skills. Licensed psychological examiners, who are trained and supervised in the assessment of development in young children by a licensed psychologist, administer the cognitive, language, and motor domains. The adaptive and social-emotional domains are rated by mothers or caregivers via questionnaires. Reliability results show strong internal consistency for this measure, with average correlations of 0.91 for the cognitive domain; 0.93 for the language domain; 0.86 for fine motor, and 0.91 for expressive and gross motor subtests. Across all ages, stability coefficients were 0.80 or higher. Validity for the BSID-III was also strong. High correlations between the BSID-III and the Wechsler Preschool and Primary Scale of Intelligence-3 ranged from 0.72 to 0.79 for cognitive and 0.71 to 0.83 for language BSID-III composite scores (Albers et al., 2006).

BSID-III was administered once when the infants were about 12 months of age (median age = 12.0 years; age range = 11.5 to 12.5 months) and then, later when the infants were about 24 months of age (median age = 24.0; age range = 23.8 to 25.9 months). The range of time between measurements was 11.5 months to 14.0 months. Mothers were present during the administration of the BSID-III. In this analysis, we focused on the composite scores for adaptive, cognitive, language, motor, and social-emotional skills at 12 and 24 months and considered these as separate, individual outcomes at each time point. The scores at 12 months and 24 months were approximately normally distributed (Fig. S1). The mean score in each of the five BSID-III domains was above 80 points, which is commonly considered a cut-off for neurodevelopmental adversities based on the BSID-III scoring system.

### 2.4. Covariate information

At the time of participant enrollment, self-reported interviews were conducted to collect socio-demographic information such as maternal age, educational attainment, and self-identified race/ethnicity. Child sex and delivery mode were obtained at the first postnatal visit. Breastfeeding initiation and cessation dates were reported by the mother at 0.5, 1, 2, 3, 4, 5, 6, 9, 12, 18 or 24 months at in-person research visits. Length of breastfeeding was calculated from these two dates (median = 7.29, range = 0.06 to 23.7).

### 2.5. Statistical analyses

Study demographics and BSID-III scores (adaptive, cognitive, language, motor, and social-emotional scaled scores) were described using frequencies or, arithmetic means and standard deviations (SDs) as appropriate. Geometric means, geometric 95 % confidence intervals (95 % CI), and selected percentiles were used to present the distributions of placental PFAS levels (PFOA, PFHxS, PFNA, PFDA, PFOS, and summed PFAS). All five placental PFAS levels were non-normally distributed and were natural log-transformed for further analyses. Violin plots were used to compare the distribution of PFAS or 12- and 24-month BSID-III scores, respectively. LOESS curves and Spearman correlations were used to examine the univariate association between each of the natural log transformed PFAS compounds and each of the BSID-III scores.

Unadjusted and adjusted linear regression models were used to assess the association between individual, natural log transformed PFAS, and each BSID-III score at 12 and 24 months. Adjusted models controlled for covariates selected *a priori* based on direct acyclic graphs (Fig. S2) and adjusted for infant sex, gestational age, maternal educational attainment, maternal age, and maternal BMI category. We considered confounding by gestational age here, even though it is commonly understood as a mediator as well because all deliveries were at-term in our study.

To examine how cumulatively increasing levels of all five placental PFAS related to neurodevelopment at 12 and 24 months, we performed mixture analyses using quantile g-computation and Bayesian kernel machine regression (BKMR). As with the linear regression models, in these analyses too, we considered each of the BSID-III composite scores at 12 and 24 months of age as separate outcomes and controlled for the same covariates as described above.

First, we used quantile g-computation to ascertain the overall effect of simultaneously increasing the placental PFAS mixture by one quartile on each of the BSID-III scores. Quantile g-computation is a parametric generalized linear model approach that implements g-computation to estimate the combined contribution of multiple exposures on a single outcome of interest (Keil et al., 2020). Here, each of the five PFAS was binned into quartiles and then included as the main exposure of interest. Positive or negative weights were assigned to each individual PFAS included in the mixture, which are based on the association and direction of effect for each individual PFAS. These weights represent the proportion of the effect that the PFAS compound contributes to the effect direction, where all positive weights add up to 1 and, all negative weights add up to 1.

Next, we assessed potential interaction and non-linear effects of placental PFAS on BSID-III scores using BKMR, which is a nonparametric approach that performs kernel machine regression to estimate a high-dimensional exposure-response function (Bobb et al., 2015; Bobb et al., 2018). Individual (or univariate) and bivariate exposure-response functions were used to investigate non-linearity and interactions between the PFAS compounds, respectively. The cumulative effect of all the PFAS levels in the placenta on a specific BSID-III score was defined as the expected difference in the BSID-III score when all PFAS compounds were set at specific quartiles and when all PFAS compounds were fixed at their 50^th^ percentile of exposure level.

Since studies have highlighted neuroendocrine disruptive and sexually dimorphic effects of PFAS on fetal development (Guidance on PFAS Exposure 2021), we conducted secondary analyses wherein we evaluated the effect of PFAS on BSID-III scores at 12 and 24 months stratified by females (*n* = 63) and males (*n* = 88) only. We performed linear regressions for individual PFAS and quantile g-computation models for mixture analyses among the female and male strata. We used quantile g-computation extension for interaction [that is, qgcomp.emm()].

We also conducted separate sensitivity analyses and assessed whether maternal self-reported race or breastfeeding duration in months or gestational age altered the observed associations between the mixtures of the five PFAS and BSID-III scores as estimated by quantile g-computation.

Lastly, since there are few studies on placental PFAS levels, we performed secondary analyses to contextualize whether different biomatrices (maternal and cord serum) for PFAS quantification would have yielded different results. We used linear regressions and quantile g-computation to estimate the associations of maternal serum PFAS levels averaged across pregnancy and cord serum PFAS levels with the BSID-III scores among those with matched maternal serum, cord serum, and placental samples (*n* = 98). We examined sex-specific effects across the biomatrices and adjusted all models for covariates as previously stated.

All statistical analyses were conducted in RStudio (version 4.3.0 or above).

## 3. Results

Our analytical sample included 151 mother-infant dyads for whom placental PFAS levels were quantified, and among these 147 had the BSID-III assessment at either 12 or 24 months of age. Most of the mothers had a college degree (70 %) and self-identified as White (86 %) (Table 1). On average, the mothers were 31 years old (SD = 3.4) at the time of enrollment. There were 63 biologically female and 88 biologically male infants, most of whom were vaginally delivered at an average gestational age of 39 weeks (SD = 0.85).

Placental PFAS levels were moderately to strongly, positively correlated with each other (Fig. S3). Of the five well-detected PFAS, the highest exposure level in the placenta was to PFOS (geometric mean = 0.38 ng/g; geometric 95 % CI = 0.34, 0.42 ng/g) and the lowest was to PFDA (geometric mean = 0.03 ng/g; geometric 95 % CI = 0.02, 0.03 ng/g) (Table 2).

**Fig. 1.**
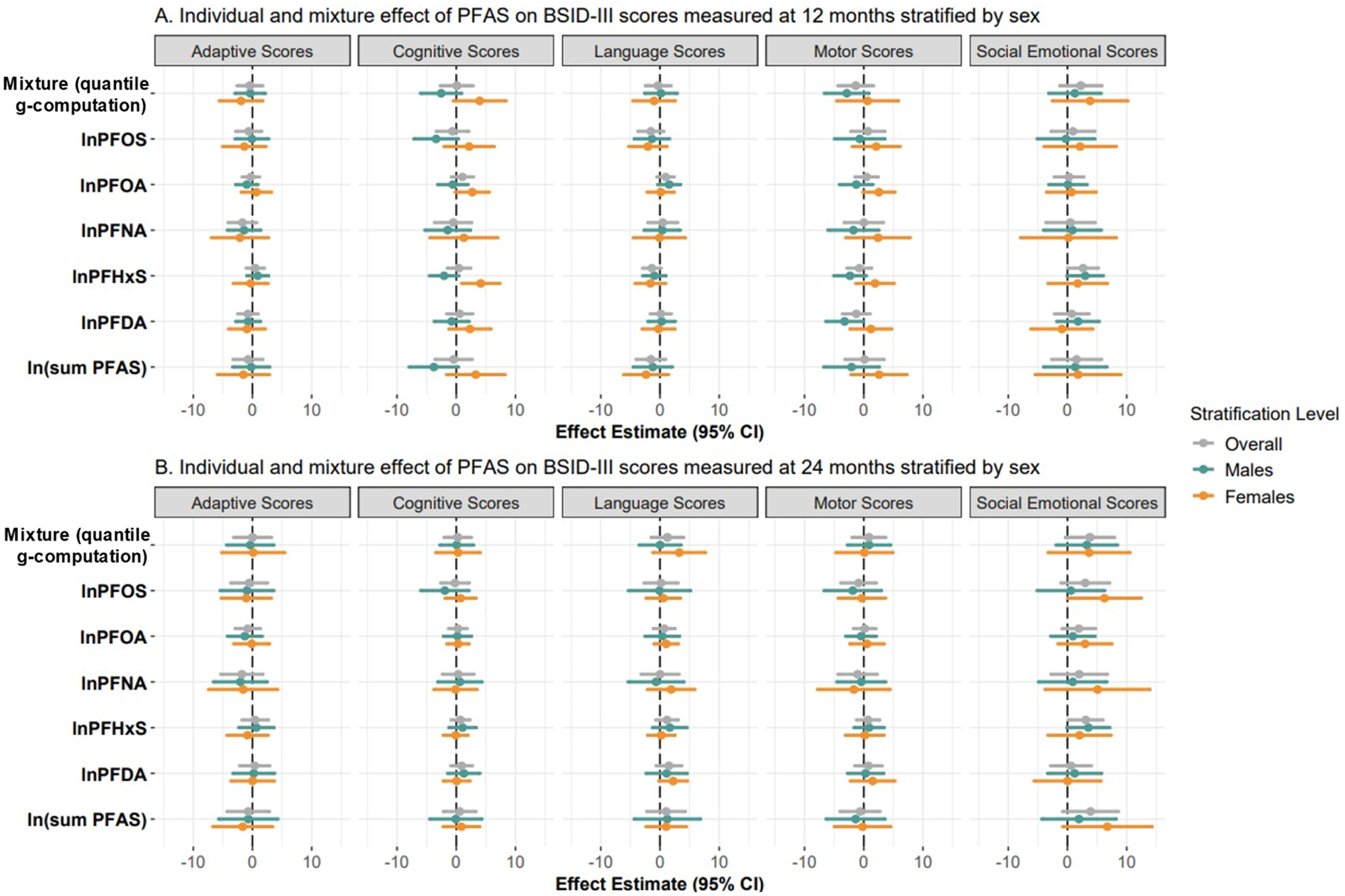
Forest plots of the association of natural log-transformed placental per- and polyfluoroalkyl substances (PFAS) levels (ng/g) individually and as a mixture, with each of the Bayley Scales of Infant and Toddler Development (BSID)-III scores at 12 months (A, top panel) and 24 months (B, bottom panel), overall and among female and male infants in the Glowing cohort, AR, USA, 2010–2014. **Note:** All overall models adjusted for infant sex, gestational age, maternal educational attainment, maternal age, and maternal body mass index category. All sex-specific models adjusted for the same covariates other than infant sex. Sample sizes for the linear regression models are detailed in Table S1–S2 while those for quantile g-computation are detailed in Table S3.

Adjusted linear regression models showed that individually, natural log-transformed PFAS were not associated with adaptive, or language scores measured at 12 or 24 months in our primary analyses (Fig. 1 and Table S2–S3). However, we identified several noteworthy trends and associations in the stratified sex-specific analyses. Higher levels of all five natural log-transformed PFAS measured in the placenta tended to be associated with lower cognitive and motor scores among males at 12 months, whereas they tended to be associated with higher cognitive and motor scores at 12 months among females. Of the five PFAS, among males, natural log-transformed placental PFDA levels were negatively associated with motor scores at 12 months (effect estimates [β] = −3.23; 95 % CI = −6.39, −0.06), while natural log-transformed placental PFOS levels were negatively associated with cognitive scores at 12 months (β = −3.39; 95 % CI = −7.12, 0.36). Among females, natural log-transformed placental PFHxS levels was positively associated with cognitive scores at 12 months (β = 4.15; 95 % CI = 0.95, 7.35) whereas, natural log-transformed placental PFOA levels were positively associated with motor skills at 12 months (β = 2.56; 95 % CI = −0.14, 5.26). Individual PFAS were associated with higher social-emotional scores at 24 months and associations were strongest among females, especially for PFOS (β = 6.24; 95 % CI = 0.03, 12.46) and PFNA (β = 5.08; 95 % CI = −3.76, 13.92). While we highlight these consistent trends in effect estimates, we acknowledge that many of the 95 % CIs do include the null.

Next, we examined how a simultaneous 25 % increase in all five placental PFAS levels was associated with BSID-III scores among females and males using quantile g-computation (Fig. 1 and Table S4). We did not identify any statistically significant mixture effects; however, there were several trends worth highlighting. Males with higher PFAS mixture levels tended to have lower cognitive (quantile g-computation effect estimate (Ψ) = −2.55; 95 % CI = −6.03, 0.92) and motor (Ψ = −2.86; 95 % CI = −6.63, 0.90) scores at 12 months, and higher social-emotional scores (Ψ = 3.26; 95 % CI = −1.92, 8.44) at 24 months. Among females, we found that those with higher PFAS had higher language scores (Ψ = 3.25; 95 % CI = −1.19, 7.68) and social-emotional scores (Ψ = 3.65; 95 % CI = −3.26, 10.55) at 24 months. Interestingly, we observed opposite directions of association for cognitive scores for males and females, with higher PFAS mixture levels related to higher cognitive (Ψ = 3.97; 95 % CI = −0.48, 8.42) scores at 12 months in females. Quantile g-computation’s partial effect indicate that PFHxS was the strongest contributor to the negative effect on cognitive scores at 12 months in males; PFDA was the strongest contributor to the positive effect on motor scores at 12 and 24 months in both females and males; and that in females, PFDA and in males, PFHxS was the greatest contributor to the negative and positive effect, respectively, on social-emotional scores at 24 months (Fig. S4). Overall, PFOS appeared to be driving the negative effects of placental PFAS mixture exposure on neurodevelopment at 12 months whereas, PFOA and PFHxS seemed to be driving the positive effects of placental PFAS mixture exposure on neurodevelopment at 24 months (Fig. S4).

**Fig. 2.**
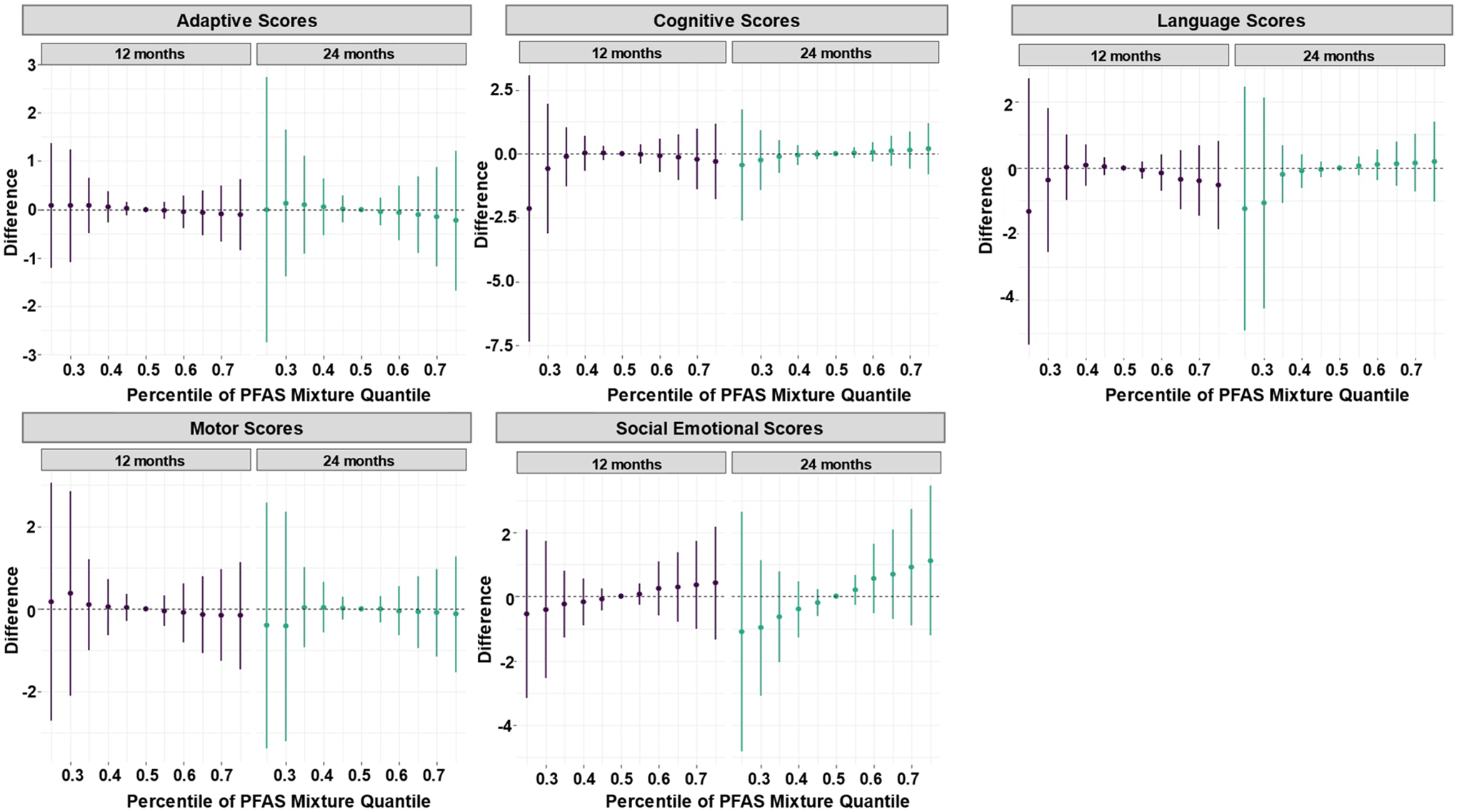
Cumulative effect and 95 % credible interval for the associations of placental per- and polyfluoroalkyl substances (PFAS) levels (ng/g) with each of the Bayley Scales of Infant and Toddler Development-III scores at 12 and 24 months, estimated using Bayesian kernel machine regression in the Glowing cohort, AR, USA, 2010–2014. **Note:** All models adjusted for infant sex, gestational age, maternal educational attainment, maternal age, and maternal body mass index category. Sample sizes for the models are the same as those for quantile g-computation and are detailed in Table S3.

BKMR also indicated that higher cumulative exposure to all five PFAS in the placenta was linked with higher social-emotional scores at 12 and 24 months (Fig. 2 and S5–S6).

We performed sensitivity analyses to examine the stability of our results. We examined how maternal self-reported race altered the observed associations of placental PFAS mixtures on neurodevelopment at 12 months and 24 months and observed some modest differences in the effect estimates (Table S5). However, considering that in our analytical dataset, only 14 % (*n* = 21) of the 151 participants self-identified as non-white, we could not examine the impact of controlling for self-identified ethnicity in the sex-stratified models. Of note, in the mixture models, the relationship between PFAS with language scores at 24 months were attenuated while the magnitude of the association for social-emotional scores increased, and all these CIs included the null. Additionally, we examined whether adjusting for breastfeeding duration, an influencer of child development and source of early-life PFAS, altered our observed associations with postnatal neurodevelopment, and we saw no large changes in the associations (Table S5). Last, while we conceptualized gestational age as a confounder, where PFAS may accumulate to higher concentrations with increasing gestational time, it may also be a mediator since PFAS may lead to shortened gestation, which is a risk factor for neurodevelopmental impairment. Thus, we also tested whether removing gestational age at birth altered our findings and found that the observed associations were similar with and without gestational age adjustments (Table S5).

To contextualize our results in the placenta, we compared PFAS levels and their associations with BSID-III scores among those with matched maternal serum, cord serum, and placental PFAS levels. Among this subset, we observed that PFAS concentrations were lowest in placenta, and highest in maternal serum (averaged across pregnancy) (Fig. S7A and Table S6). Despite differences in average concentrations, maternal serum and placental PFAS levels were more strongly correlated with each other overall, compared to cord serum. Maternal serum and placenta concentrations of PFOS, PFOA, PFNA, and PFHxS were very highly correlated between tissues (r between 0.67 and 0.83; all *p* <0.05), while PFDA was only modestly correlated (0.38, *p* < 0.05), likely due to lower detection rate of PFDA (Fig. S7B).

In linear regression and quantile g-computation models, we observed mostly small to no associations across the biomatrices, and where there were trends in association, the directionality of the association was mostly consistent across the biomatrices (e.g., maternal serum, cord serum, and placenta PFHxS levels were all negatively associated with language scores at 12 months among females). We observed some apparent unique biomatrix relationships, where cord serum associations were larger for mixture effects and for the individual associations with PFDA, but they were accompanied by very wide confidence intervals, most likely due to the low detection rate of PFDA in cord serum. We also found that among females, higher cord serum PFOA was associated with lower language scores at 12 months (β = −6.41; 95 % CI = −10.51, −2.30) and lower adaptive scores at 24 months (β = −9.76; 95 % CI = −15.73, −3.80); the same directions of effect were observed for maternal serum and placental PFOA, but only cord serum yielded confidence intervals that excluded the null. Overall, we observed that sex-specific effects were most apparent when placental PFAS or cord serum were modeled as the exposure (Fig. S8–S9).

## 4. Discussion

We examined the associations between placental PFAS levels on five neurodevelopmental outcomes measured in 12- and 24-month-old children from central Arkansas, US. Overall, we observed that placental levels of PFOA, PFHxS, PFNA, PFDA, and PFOS, individually and as a mixture, had different associations with each of the neurodevelopmental outcomes at 12 and 24 months with some sex-specific associations among female and male infants. However, most of the observed associations were imprecise, which may be reflective of our modest sample size. Our results underscore the complex relationship between placental exposure to multiple PFAS compounds and postnatal neurodevelopment in young children.

While some of our observed associations align with our hypothesis that PFAS would negatively impact BSID-III scores, several of our findings contrasted this hypothesis. To our knowledge, no other studies have examined whether placental PFAS levels are associated with neurodevelopment. Thus, while our work fills an important gap, we cannot make direct comparisons to the published literature. However, our results do somewhat align with the mixed results reported by past epidemiological research on the relationship between maternal blood or cord blood PFAS levels in relation to neurodevelopment.

We found that placental PFAS levels did not influence adaptive skills at 12 or 24 months. Other studies evaluating this relationship have reported statistically significant negative (Yao et al., 2022) or positive (Luo et al., 2022) effects of prenatal PFAS on adaptive skills. For cognitive and motor skills, we observed sex-specific associations with placental PFAS, individually and as a mixture. We found that higher exposures were associated with lower scores among males but higher scores among females and that PFNA, PFOA, PFDA, and PFOS drove the relationships. Some of our findings of sex-specific associations align with those observed in the Sheyang Mini Birth Cohort study wherein cord blood PFDA was also negatively associated with motor skills at 1 year among males, and cord blood PFOA was also positively associated with motor skills at 1 year among females (Zhang et al., 2023). Also, among 302 mother-children pairs from New York City, higher levels of cord blood PFOA and PFNA were each associated with lower scores on the Psychomotor Development Index (which assesses fine and gross motor skills) at 1 year for males (Spratlen et al., 2020). Other epidemiological studies also suggested PFOS (Luo et al., 2022; Reardon et al., 2023; Chen et al., 2013; Gao et al., 2024; Li et al., 2023), PFHxS (Luo et al., 2022; Yao et al., 2022), PFNA (Luo et al., 2022; Reardon et al., 2023), PFOA (Oh et al., 2021), and PFDA’s (Reardon et al., 2023; Li et al., 2023) negative association with gross motor skills and PFOS (Reardon et al., 2023), PFHxS (Luo et al., 2022; Reardon et al., 2023; Gao et al., 2024), PFNA (Luo et al., 2022; Reardon et al., 2023), and PFOA’s (Luo et al., 2022; Reardon et al., 2023; Chen et al., 2013; Gao et al., 2024) positive association with cognition but more broadly in all children and so, further evaluations of the drivers of the sex-specific effects are needed. We observed more prominent associations on cognitive and motor skills at 12 months than 24 months in our analysis. The impact of PFAS on neurodevelopmental trajectories has not been well examined, with only one study examining cord blood PFAS and neurodevelopment at 2, 6, 12, and 24 months of age reporting sexual dimorphism in the association (Zhou et al., 2023). More research is warranted to assess the reliability and robustness of these findings.

We found that only among females, placental PFAS, especially as a mixture, trended towards a positive association with language skills at 24 months, but these associations were not statistically significant. This is consistent with non-sex-specific findings for maternal blood PFOA, PFHxS, PFDA, and PFOS in a Canadian cohort (Reardon et al., 2023) but is contradictory with some other cohorts’ results. For example, in MARBLES, a cohort over-selected for infants with autism spectrum, while maternal blood PFOA was linked with significantly reduced receptive and expressive language at 24 months, maternal blood PFOS was linked with higher scores of these skills, and maternal blood PFHxS, PFNA, and PFDA were not associated at all (Oh et al., 2021). Among children from Japan, cord blood PFOA levels were linked with lower receptive language skills at 10 months whereas, PFOS levels were linked with significantly higher expressive language at 24 months (Oh et al., 2022). In a birth cohort from China, maternal serum levels of 10 PFAS cumulatively associated with significantly lower language skills (Luo et al., 2022).

In our study, among females and males, higher placental PFAS was linked with higher social-emotional skills at 12 and 24 months, a finding that aligns with another study examining cord blood PFAS and neurodevelopmental trajectories (Zhou et al., 2023). However, more rigorous human and animal studies are needed to inquire about the biological or developmental pathways that underlie whether and how PFAS impact social-emotional repertoires. Our findings with social-emotional skills seemed to be more prominent at 24 months than 12 months in our study. Past studies report mixed results about the associations at 24 months. In the Shanghai Maternal-Child Pairs cohort, cumulative exposure to 10 cord blood PFAS was linked with non-statistically significant increase in personal-social skills at 12 and 24 months among females but a not significant decrease in personal-social skills at 12 and 24 months among males (Zhou et al., 2023). Among 2-year-old Taiwanese children, cord blood PFOS was negatively associated with social and self-help skills and on the other hand, cord blood PFOA was linked with higher social skills and lower self-help skills (Chen et al., 2013). In contrast, analyses from the Shanghai Birth Cohort found no change in social-emotional skills at 24 months upon simultaneous increase in a maternal blood PFAS mixture, but did observe individual associations for maternal blood PFOA, PFOS, PFNA, or PFHxS with lower social-emotional scores (Luo et al., 2022).

While we observed that PFAS has some positive or seemingly “protective” relationships with neurodevelopment, these associations may not necessarily mean faster neurodevelopment or improved outcomes and improved quality of life for the child. Likewise, our null associations also do not indicate that placental PFAS are not associated with any neurodevelopmental vulnerabilities or related childhood adversities in our study as BSID is not a diagnostic tool; and we only measured BSID twice and did not analyze neurodevelopmental domains not assessed by the BSID.

We performed secondary analyses to compare the relationships between PFAS and BSID scores across three biomatrices (placenta, maternal serum, cord serum), in a subset of participants with biomatrix-matched samples. We found that associations (and lack of associations) were mostly consistent across biomatrices. However, sex-specific effects were most notable in associations with placental PFAS compared to other matrices. Thus, the placenta may be an important biomatrix for considering sex-specific effects. The biological rationale for this might lie in the fact that the placenta has sexual dimorphic molecular mechanisms geared to protecting the fetus and supporting fetal development in a sex-specific manner (Kalisch-Smith et al., 2017). Comprehensive epidemiological research comparing biomatrix-specific PFAS levels, their associations and their predictive power for child health is needed. While this work is ongoing in our cohort, similar studies are needed in other cohorts as well.

The profile of PFAS exposure in our study sample was similar to what other studies have reported. While no other studies have compared placental PFAS to neurodevelopment, several cohorts that have measured PFAS in placenta, including two different cohorts of ~120 women in North Carolina (Bangma et al., 2020; Hall et al., 2022), a cohort of 78 women in Denmark (Mamsen et al., 2019), and a cohort 50 women in southern Israel (Groisman et al., 2023). For all of these, the greatest concentration level in placentae was for PFOS and there were lower levels of PFDA, PFNA, and other PFAS. In our cohort and other US based cohorts that were enrolled at a similar time, PFOS was a notable contributor to the placental PFAS’s exposure profile. This also aligns with the PFAS exposures assessments from the NHANES from 1999 to 2008 (Kato et al., 2011) and 2015 to 2016 (Schultz et al., 2023), which reported that maternal serum PFOS was present at higher levels as compared to other PFAS. Additionally, when we compare our PFAS levels directly to other US cohorts that enrolled participants at a similar time, our levels are comparable. One of the North Carolina cohorts reported similar median levels of placental PFOS (0.48 ng/g) and PFHxS (0.067 ng/g) compared to our samples (PFOS = 0.43 ng/g and PFHxS = 0.05 ng/g), but they did not have enough values above the LOD for PFNA, PFDA, or PFOA to compare to our cohort (Bangma et al., 2020). Alternatively, another North Carolina cohort had slightly higher median levels of PFOS (0.95 ng/g) and PFOA (0.27 ng/g), but with similar median levels of PFNA (0.11 ng/g) and PFDA (0.06 ng/g), and low detection of PFHxS (Hall et al., 2022). Further, the PFAS concentrations in placental tissue have limited comparability to the PFAS levels measured in blood, plasma, or serum due to matrix differences, making it difficult to interpret and translate findings that utilize different biomatrices for quantification.

Our results should be interpreted in view of our analytical limitations. Foremost, our inferences are limited to placenta as a biomatrix and select PFAS compounds. Our study only focuses on PFAS well-detected in placenta, and all of these happened to be long chained perfluoroalkyl carboxylates or sulfonates (Buck et al., 2011). This could be due to low levels of exposure to shorter chained PFAS in our study or as demonstrated by others (Zhang et al., 2013), differences in the transfer and partition of PFAS across placenta, maternal and cord blood based on PFAS chain length. Our study relied on the BSID-III assessment and scoring based on the new BSID-IV may not align with our results (Anderson and Burnett, 2017; Aylward, 2020). Our results may also be impacted by residual confounding. Importantly, the interpretability of our results is limited to healthy populations. As with many epidemiological birth cohort studies, our analysis is subject to live birth bias which may have contributed to some of our observed positive associations. Also, our results may not be generalizable to other diverse populations of the US who may experience different types and doses of PFAS exposure. Notably, much of our analytical subset were term births and our study excluded adverse pathologies (e.g., preeclampsia, pre-term birth, gestational hypertension, or gestational diabetes). These factors could have introduced selection bias in our study and may have biased our results towards the null. We also tested for sex-specific associations, with particularly interesting trends observed for cognitive scores, however, our sex-stratified sample sizes were quite small and larger sample sizes are needed to robustly investigate sex-specific associations. Furthermore, the synergistic or antagonistic effect of pre- and post-natal co-exposure to other PFAS compounds (e.g., breastmilk PFAS, formula and tap water, and newer PFASs) and other environmental neurotoxicants (such as pesticides, heavy metals etc.) on neurodevelopment should be explored.

There are also many strengths of our analysis. First, our exposure assessment scenario and approach provide unique insights into PFAS’ developmental neurotoxicity from an epidemiological perspective. We measured PFAS levels in human placental tissue, which may be a more proximal *in utero* biomatrix than maternal serum and the placenta is the direct moderator of the *in utero* environment. By using gold standard quantification techniques (HPLC-MS/MS), we were able to achieve high detection of placental PFAS. We examined the relation between placental PFAS and neurodevelopment at two postnatal time points using three statistical approaches: linear regression, quantile g-computation, and BKMR, which informed us about the individual and dose-dependent, joint, and non-linear relationships. We observed some distinct, opposing sexually dimorphic associations of PFAS with neurodevelopment, even though our analysis was limited by strata-specific sample size and sparse data. Our observation of sex-dependent associations emphasizes the importance of testing for these, and that not accounting for sex-differences could lead us to underestimate the risk posed by placental PFAS levels for neurodevelopment or other outcomes. We also observed consistency in the associations across our different analytic approaches, providing robustness to our results.

## 5. Conclusion

In a prospective cohort of 151 mother-infant dyads from Arkansas, US, we found that placental PFAS levels were mostly not associated with neurodevelopment outcomes among 12- and 24-month-old infants and there were subtle sex-specific associations. Placental PFAS as a mixture was associated with lower cognitive and motor scores at 12 months among males and higher language scores among females. Placental PFAS as a mixture was associated with higher social-emotional scores at 24 months among males and females. However, for all these associations, the magnitude was small and their clinical significance should be further investigated. Future studies should investigate the differential effects of legacy and new PFAS, and their combined effect on the rate and timing of neurodevelopmental trajectories across wider and multiple windows.

## Supplementary Material

Supplementary Material

Supplementary material associated with this article can be found, in the online version, at doi:10.1016/j.envadv.2025.100650.

## Figures and Tables

**Table 1:** Descriptive statistics of demographics in the Glowing cohort, AR, USA, 2010–2014.

	Frequency (%) or Mean (SD) in the analytical subset			Frequency (%) or Mean (SD) of infants in the Glowing cohort		
	Female (*n* = 63)	Male (*n* = 88)	Overall (*n* = 151)	Female (*n* = 90)	Male (*n* = 123)	Overall (*n* = 213)
**Maternal Age at Enrollment (years)**						
Mean (SD)	31 (3.4)	30 (3.5)	31 (3.4)	30 (3.5)	29 (3.5)	30 (3.5)
**Maternal Education**						
College Degree	45 (71 %)	60 (68 %)	105 (70 %)	60 (67 %)	81 (66 %)	141 (66 %)
No College Degree	18 (29 %)	28 (32 %)	46 (30 %)	30 (33 %)	42 (34 %)	72 (34 %)
**Maternal Self-Identified Race** *						
White	54 (86 %)	76 (86 %)	130 (86 %)	76 (84 %)	109 (90 %)	185 (88 %)
Black and other races	9 (14 %)	12 (14 %)	21 (14 %)	14 (16 %)	12 (10 %)	26 (12 %)
**Maternal Body Mass Index category**						
18.5 to 24.9 kg/m^2^	27 (43 %)	47 (53 %)	74 (49 %)	41 (46 %)	58 (47 %)	99 (46 %)
25 to 35 kg/m^2^	36 (57 %)	41 (47 %)	77 (51 %)	49 (54 %)	65 (53 %)	114 (54 %)
**Gestational Age at delivery (weeks)**						
Mean (SD)	39 (0.79)	39 (0.89)	39 (0.85)	39 (1.03)	39 (1.06)	39 (1.04)
**Delivery Mode**						
C-Section	25 (40 %)	26 (30 %)	51 (34 %)	37 (41 %)	36 (29 %)	73 (34 %)
Vaginal	38 (60 %)	62 (70 %)	100 (66 %)	53 (59 %)	87 (71 %)	140 (66 %)
**Infant Race** *						
White	51 (81 %)	74 (84 %)	125 (83 %)	74 (82 %)	105 (85 %)	179 (84 %)
Black and other races	12 (19 %)	14 (16 %)	26 (17 %)	16 (18 %)	18 (15 %)	34 (16 %)

Note:

* Other races in our cohort include Black, Asian, American Indian or Alaska Native, Multiple races, and non-reports and their specific statistics and percentages have not been reported due to low frequency categories and to protect confidentiality.

**Abbreviations:** SD, standard deviation; n, sample size.

**Table 2:** Description of placental levels of per- and polyfluoroalkyl substances (PFAS; ng/g) and Bayley Scales of Infant and Toddler Development (BSID)-III scores in the Glowing cohort, AR, USA, 2010–2014.

	n	% Above LOD	Mean*	95 %CI or ±Standard Deviation*	Percentiles				
					5 %	25 %	50 %	75 %	95 %
**Placental PFAS (ng/g)**									
Placental PFOA	151	74.8 %	0.05	0.04, 0.06	<LOD	<LOD	0.07	0.11	0.24
Placental PFHxS	151	83.4 %	0.05	0.04, 0.06	<LOD	0.04	0.05	0.08	0.23
Placental PFNA	151	98.0 %	0.05	0.04, 0.05	0.02	0.03	0.05	0.08	0.12
Placental PFDA	151	68.9 %	0.03	0.02, 0.03	<LOD	<LOD	0.04	0.05	0.07
Placental PFOS	151	98.7 %	0.38	0.34, 0.42	0.12	0.26	0.43	0.58	0.97
Placental summed PFAS	151	-	0.61	0.55, 0.67	0.23	0.4	0.63	0.9	1.66
**Bayley Scales of Infant and Toddler Development (BSID)-III scores**									
Adaptive Scores									
12 months	130	-	93.6	±8.88	79	87	93	99	108
24 months	124	-	96.3	±12.6	76	89	98	104.25	114
Cognitive Scores									
12 months	127	-	106	±11.2	86.5	100	105	110	125
24 months	125	-	97.2	±9.23	81	90	100	105	110
Language Scores									
12 months	125	-	93.3	±8.81	79	89	94	97	108.4
24 months	118	-	98.8	±10.7	83	91	100	103	118
Motor Scores									
12 months	126	-	102	±11.9	85	94	100	110	121
24 months	97	-	102	±10	88	94	100	110	118.60
Social Emotional Scores									
12 months	135	-	106	±15	85	95	105	112.5	140
24 months	123	-	113	±16.8	90	100	110	130	140

Note:

* For placental PFAS, geometric mean and geometric 95 % confidence interval are reported, whereas for BSID-III, arithmetic mean and arithmetic standard deviation are reported.

**Abbreviations:** CI, confidence interval; LOD, limit of detection; n, sample size.

## Data Availability

Data and analytical code is available upon reasonable request to the corresponding author.
