# Supplementary material for: Individual and mixture associations of placental per- and polyfluoroalkyl substance with neurodevelopment at 12 and 24 months of age
Source: Environ Adv. Author manuscript; Available in PMC 2025 Sep 25. (PMC12453629; doi:10.1016/j.envadv.2025.100650)
Supplement: Supplementary Material [file NIHMS2094499-supplement-Supplementary_Material.docx]

**Supplementary File for** Individual and mixture associations of placental per- and polyfluoroalkyl substance with neurodevelopment at 12 and 24 months of age

**Figure S1.** Violin plots showing the distribution of each of the Bayley Scales of Infant and Toddler Development (BSID)-III scores at 12 and 24 months among females and males only in the Glowing cohort, AR, USA, 2010-2014.


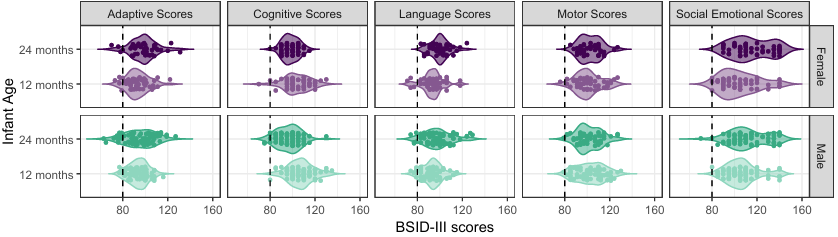


**Note**: The dotted line shows the BSID-III score of 80 which is commonly considered as a cut-off for neurodevelopmental adversities based on the BSID-III scoring system.

**Figure S2.** Direct acyclic graph of the association between placental per- and polyfluoroalkyl substances (PFAS) and Bayley Scales of Infant and Toddler Development (BSID)-III scores at 12 and 24 months in the Glowing cohort, AR, USA 2010-2014.


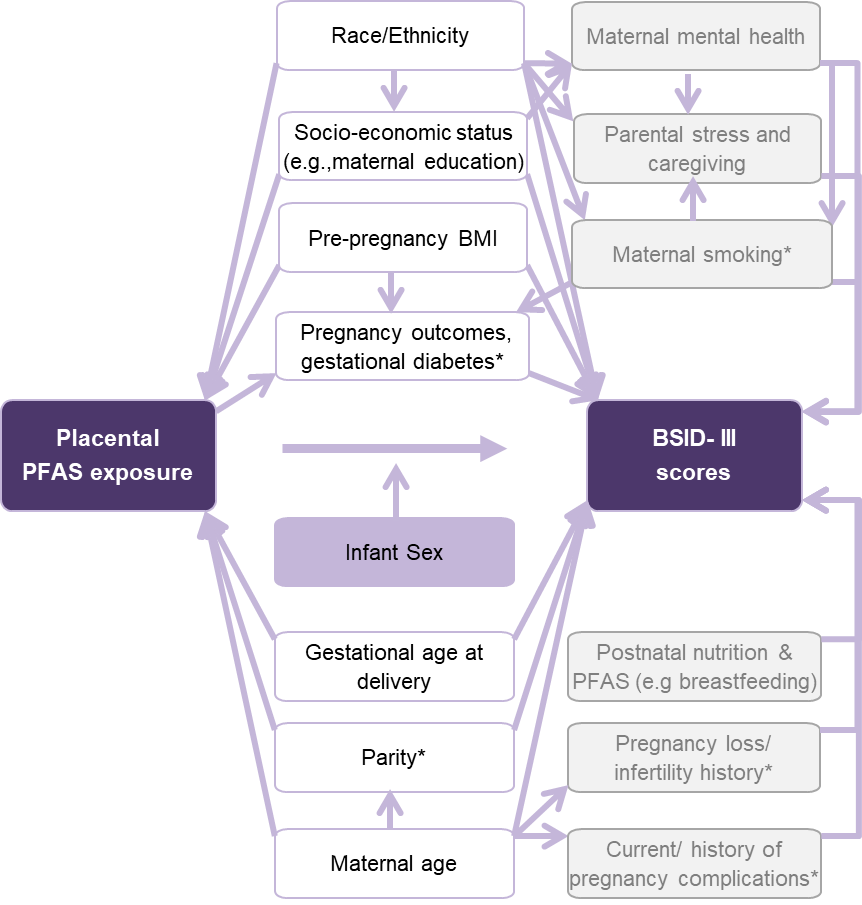


**Note:** Some of these factors (in grey) were not measured in our cohort but are relevant considerations of the associations. * denotes variables that were part of this cohort’s exclusion criteria.

**Figure S3.** Spearman correlation matrix between natural log-transformed placental per- and polyfluoroalkyl substances (PFAS) levels (ng/g) in the Glowing cohort, AR, USA, 2010-2014.

**
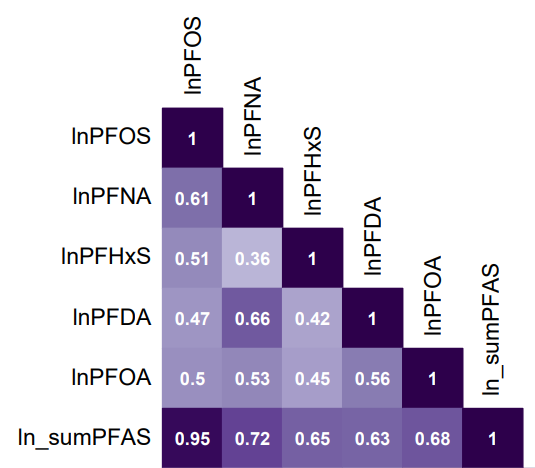
**

**Figure S4.** Heatmap showing the positive and negative weight of each partial effect of the five placental per- and polyfluoroalkyl substances (PFAS) levels (ng/g) on each of the Bayley Scales of Infant and Toddler Development (BSID)-III scores at 12 months (A, top panel) and 24 months (B, bottom panel), estimated using quantile g-computation overall, and among females and males only in the Glowing cohort, AR, USA, 2010-2014.
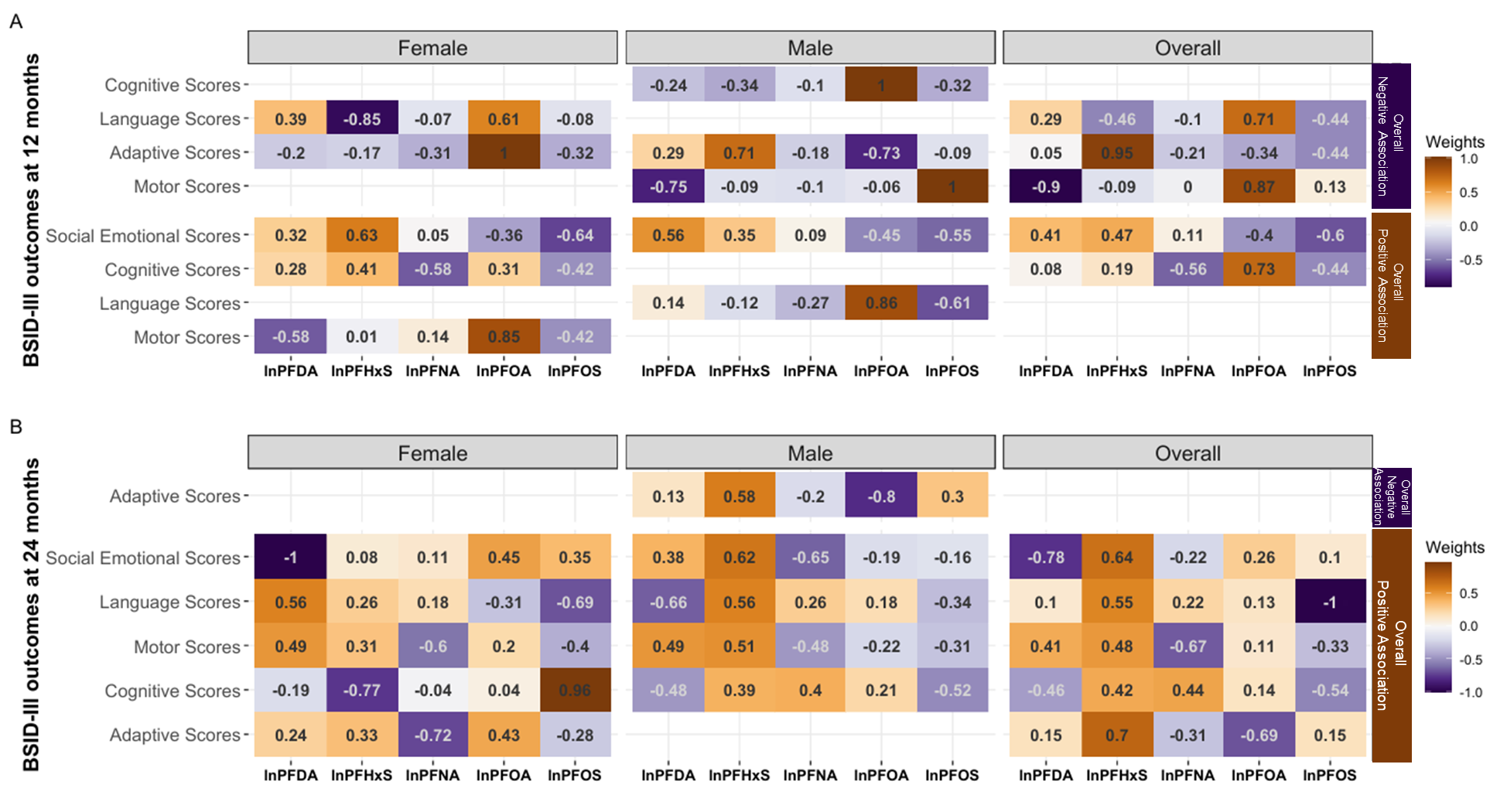


**Note:** All overall models adjusted for infant sex, gestational age, maternal educational attainment, maternal age, and maternal body mass index category. All sex-specific models adjusted for the same covariates other than infant sex. In a regression model (that is, each row), all partial weights in the positive direction (shown in orange) sum to 1, and all partial weights in the negative direction (shown in purple) sum to 1. Each partial weight shows the percent of contribution to the effect size in an effect direction. Negative and positive weights should not be compared against each other.

**Figure S5.** Individual exposure–response relation and 95% credible intervals for the change in each Bayley Scales of Infant and Toddler Development (BSID)-III scores at 12 months from a single placental per- and polyfluoroalkyl substances (PFAS) level (ng/g) while fixing all the remaining PFAS exposures in the mixture at their median concentration level, estimated using Bayesian kernel machine regression (BKMR) in the Glowing cohort, AR, USA, 2010-2014.


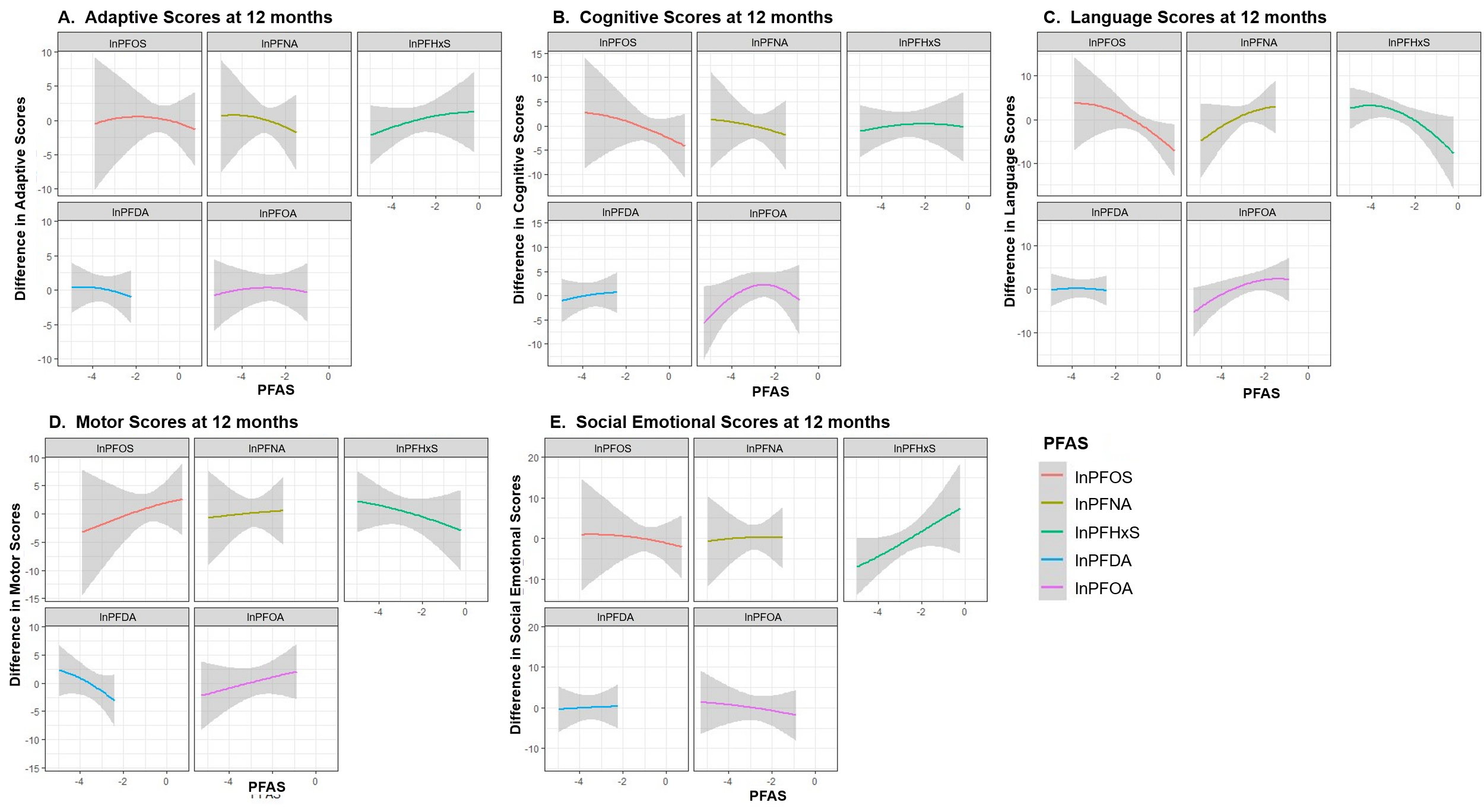


**Note:** All models adjusted for infant sex, gestational age, maternal educational attainment, maternal age, and maternal body mass index category.

**Figure S6**. Individual exposure–response relation and 95% credible intervals for the change in each Bayley Scales of Infant and Toddler Development (BSID)-III scores at 24 months for a single placental per- and polyfluoroalkyl substances (PFAS) level (ng/g) while fixing all the remaining PFAS exposures in the mixture at their median concentration level, estimated using Bayesian kernel machine regression (BKMR) in the Glowing cohort, AR, USA, 2010-2014.


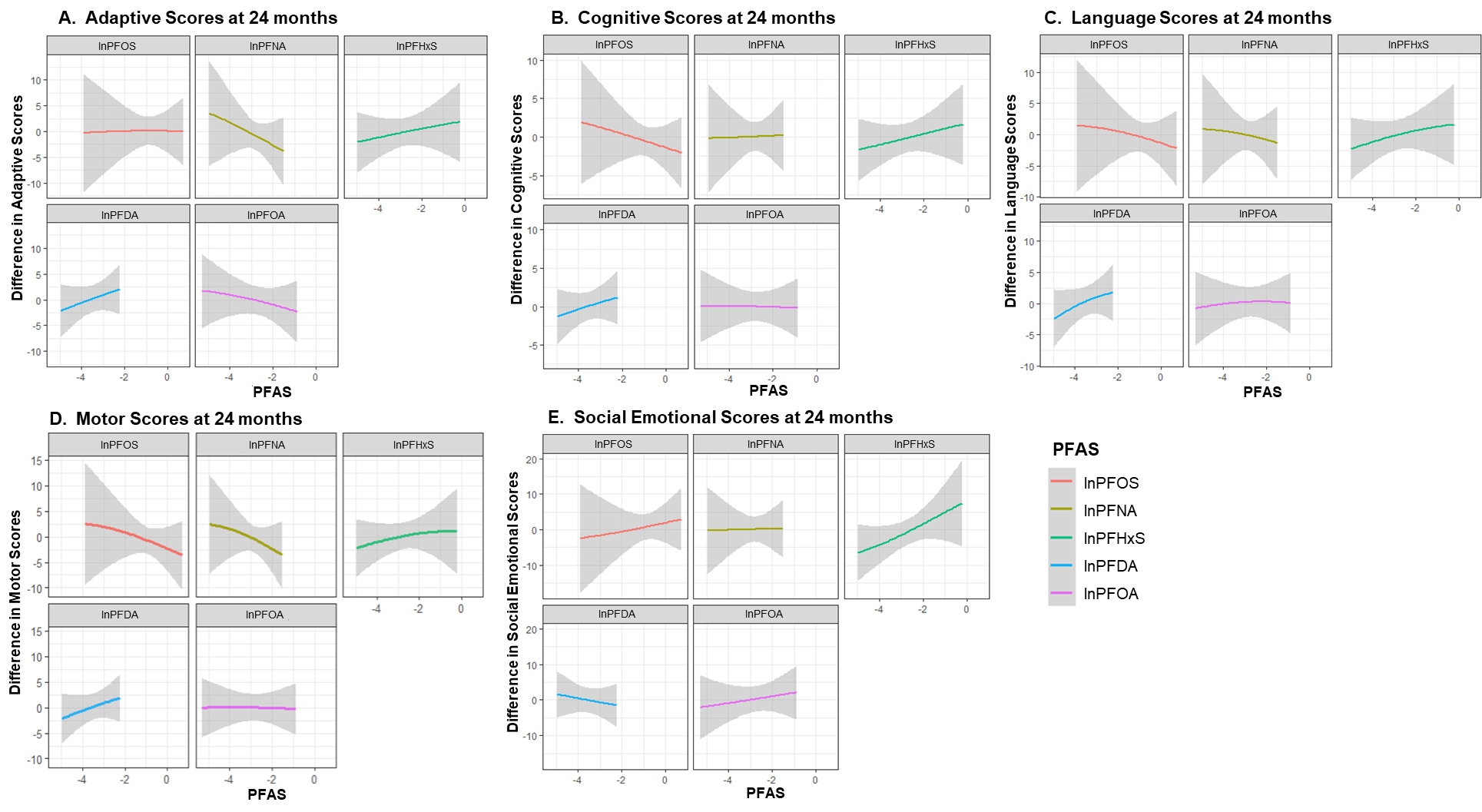


**Note:** All models adjusted for infant sex, gestational age, maternal educational attainment, maternal age, and maternal body mass index category.

**Figure S7.** Violin plots showing the biomatrix-specific distribution (A) and Spearman correlation matrix (B) of natural log-transformed per- and polyfluoroalkyl substances (PFAS) levels among those with matched maternal serum (averaged across pregnancy), cord serum and placenta samples in the Glowing cohort, AR, USA, 2010-2014.

1. **Violin Plots**


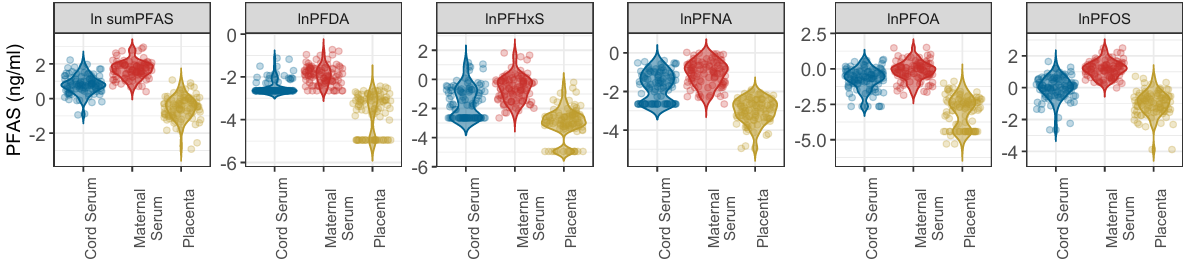


1. **Correlation Matrix**


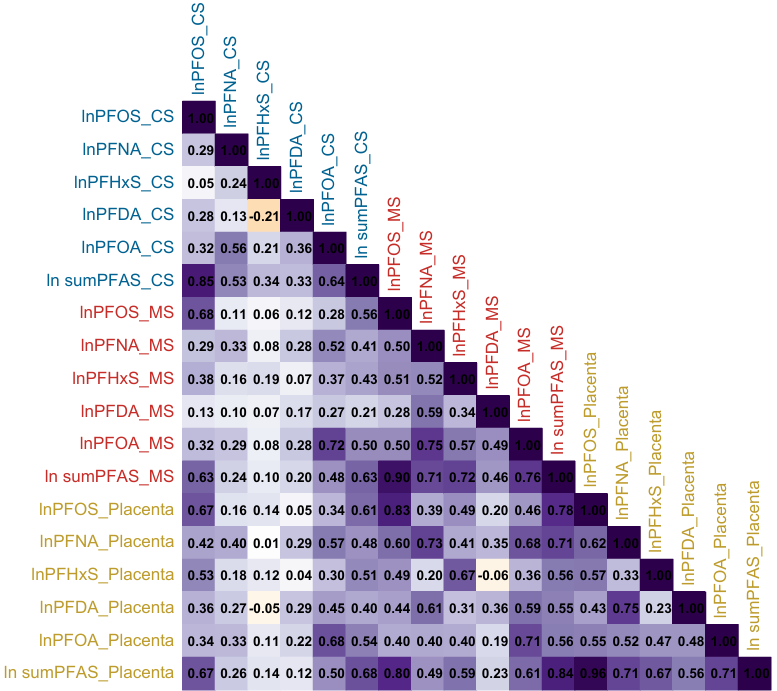


**Abbreviations:** CS, cord serum; ln, natural log; MS, maternal serum.

**Figure S8.** Forest plots of biomatrix-specific associations of natural log-transformed per- and polyfluoroalkyl substances (PFAS) levels (ng/g) individually and as a mixture, with each of the Bayley Scales of Infant and Toddler Development (BSID)-III scores at 12 months, overall and among female and male infants in the Glowing cohort, AR, USA, 2010-2014.


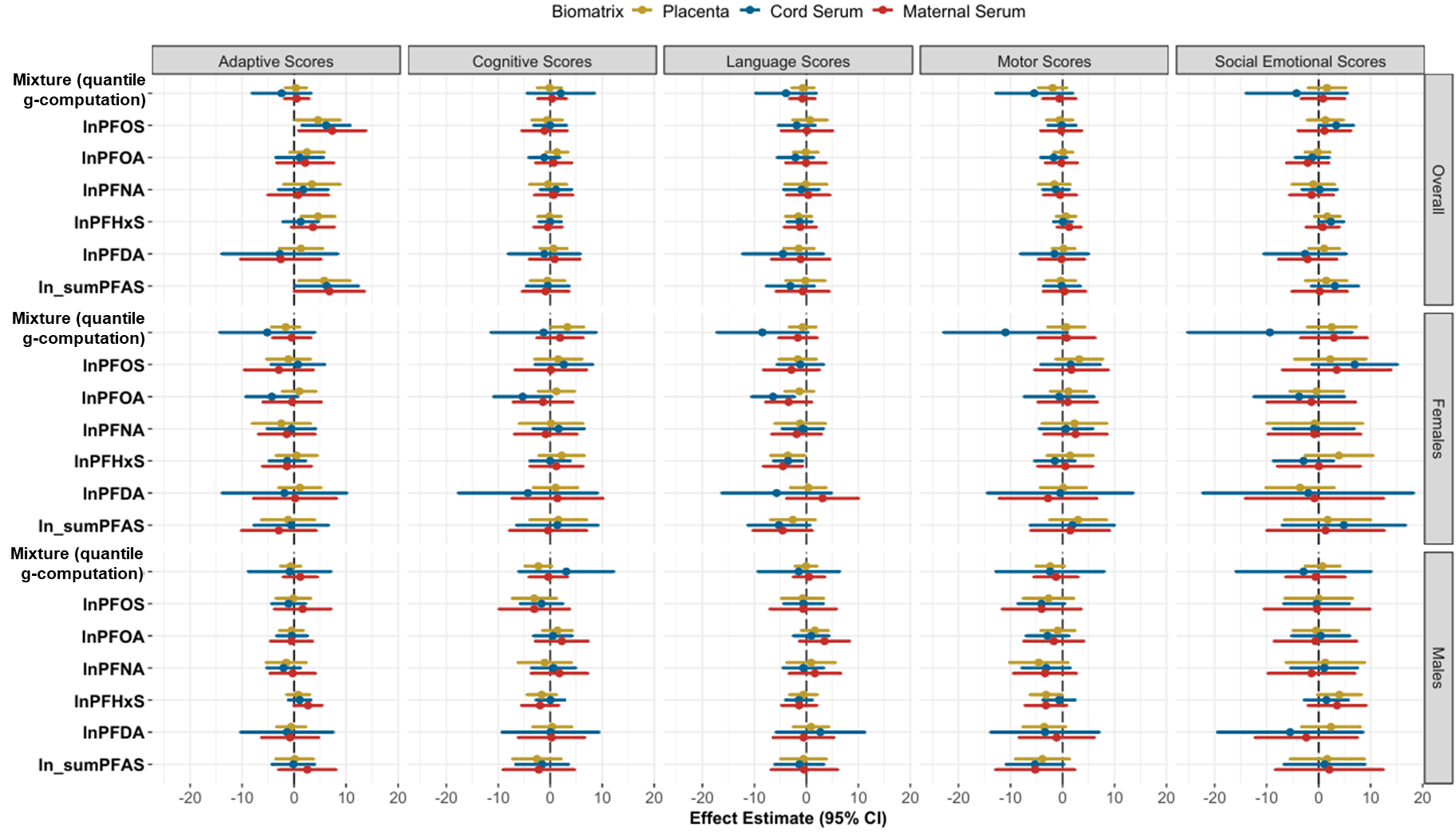


**Note:** All overall models adjusted for infant sex, gestational age, maternal educational attainment, maternal age, and maternal body mass index category. All sex-specific models adjusted for the same covariates other than infant sex. Sample size is restricted to those with matched maternal serum, cord serum and placental samples.

**Figure S9.** Forest plots of biomatrix-specific associations of natural log-transformed per- and polyfluoroalkyl substances (PFAS) levels (ng/g) individually and as a mixture, with each of the Bayley Scales of Infant and Toddler Development (BSID)-III scores at 24 months, overall and among female and male infants in the Glowing cohort, AR, USA, 2010-2014.

**
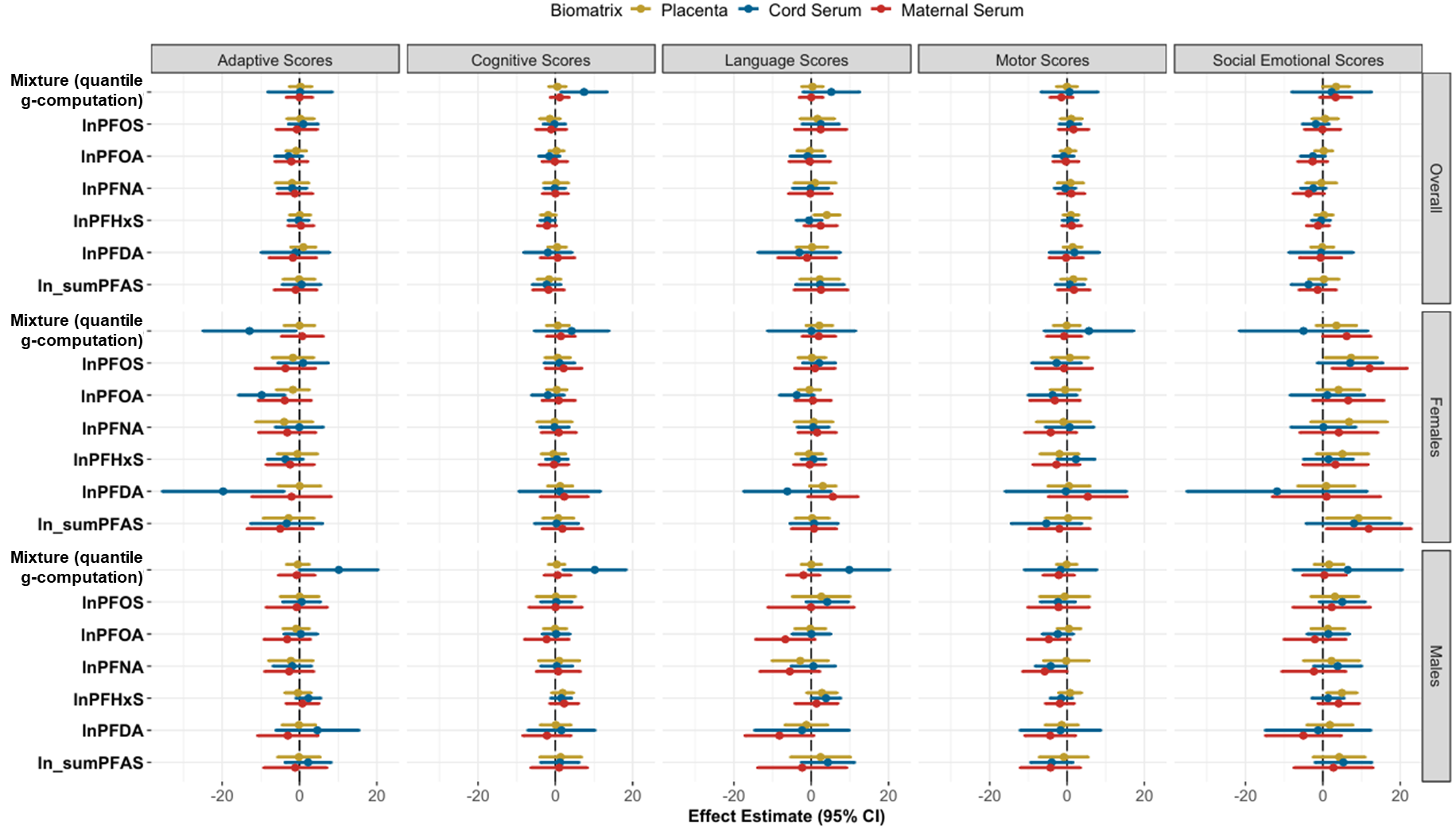
**

**Note:** All overall models adjusted for infant sex, gestational age, maternal educational attainment, maternal age, and maternal body mass index category. All sex-specific models adjusted for the same covariates other than infant sex. Sample size is restricted to those with matched maternal serum, cord serum and placental samples.

**Table S1.** Relative recoveries of each per- and polyfluoroalkyl substances (PFAS) at two different concentrations in the Glowing cohort, AR, USA, 2010-2014.

| **PFAS** | **Relative Recoveries (SD)** | |
| --- | --- | --- |
|  | **0.5 ng/mL** | **5 ng/mL** |
| **PFPeA** | 95.4 ± 7.9 | 96.5 ± 8.7 |
| **PFHxA** | 92.3 ± 1.8 | 101.3 ± 7.3 |
| **HFPO-DA** | 84.7 ± 4.2 | 87.2 ± 7.5 |
| **PFBS** | 91.7 ± 6.4 | 101.3 ± 7.3 |
| **PFHpA** | 97.0 ± 3.3 | 102.0 ± 6.6 |
| **PFOA** | 110.5 ± 2.8 | 109.6 ± 3.2 |
| **PFHxS** | 92.9 ± 10.8 | 95.1 ± 4.0 |
| **PFNA** | 91.2 ± 4.1 | 93.6 ± 4.4 |
| **PFHpS** | 116.3 ± 9.0 | 110.0 ± 8.0 |
| **PFDA** | 90.9 ± 7.9 | 96.3 ± 5.9 |
| **PFOS** | 98.0 ± 13.3 | 90.8 ± 10.3 |
| **PFUnDA** | 90.3 ± 10.6 | 104.7 ± 14.2 |
| **MePFOSAA** | 114.7 ± 3.2 | 82.3 ± 8.1 |
| **PFDoDA** | 93.9 ± 4.8 | 96.7 ± 11.3 |
| **PFDS** | 113.8 ± 3.3 | 100.5 ± 13.6 |
| **EtPFOSAA** | 114.3 ± 12.7 | 98.0 ± 13.7 |
| **PFOSA** | 97.4 ± 9.2 | 93.8 ± 9.4 |

**Table S2.** Unadjusted and adjusted linear regression summary of the association between individual placental per- and polyfluoroalkyl substances (PFAS) levels (ng/g) and each of the Bayley Scales of Infant and Toddler Development (BSID)-III scores at 12 months in the Glowing cohort, AR, USA, 2010-2014.

|  |  |  | **lnPFOS** | **lnPFNA** | | **lnPFHxS** | | **lnPFDA** | **lnPFOA** | **ln(sum PFAS)** | |
| --- | --- | --- | --- | --- | --- | --- | --- | --- | --- | --- | --- |
|  |  | **n** | β (95%CI) | β (95%CI) | | β (95%CI) | | β (95%CI) | β (95%CI) | β (95%CI) | |
| **Unadjusted Linear Models for 12 months** | | | | | | | | | | | |
| **Adaptive skills** | Overall | 130 | -0.78 (-2.97, 1.40) | -1.69 (-4.07, 0.69) | | 0.53 (-0.98, 2.04) | | -0.75 (-2.44, 0.93) | -0.16 (-1.62, 1.30) | -0.80 (-3.30, 1.70) | |
|  | Males | 78 | -0.35 (-3.17, 2.49) | -1.49 (-4.23, 1.26) | | 0.83 (-0.94, 2.61) | | -0.79 (-2.86, 1.29) | -0.71 (-2.55, 1.15) | -0.31 (-3.43, 2.81) | |
|  | Females | 52 | -1.31 (-4.87, 2.27) | -2.23 (-6.91, 2.45) | | -0.04 (-2.86, 2.79) | | -0.8 (-3.79, 2.19) | 0.48 (-1.98, 2.96) | -1.52 (-5.8, 2.77) | |
| **Cognitive skills** | Overall | 127 | -0.66 (-3.41, 2.09) | -0.49 (-3.60, 2.62) | | 0.82 (-1.13, 2.76) | | 0.87 (-1.31, 3.04) | 1.04 (-0.82, 2.90) | -0.33 (-3.49, 2.83) | |
|  | Males | 74 | -3.49 (-7.24, 0.26) | -1.65 (-5.42, 2.12) | | -1.53 (-3.98, 0.92) | | -0.76 (-3.66, 2.15) | -0.64 (-3.18, 1.9) | -3.75 (-7.93, 0.43) | |
|  | Females | 53 | 2.12 (-1.89, 6.14) | 1.39 (-4.07, 6.87) | | 4.1 (1.05, 7.14) | | 2.33 (-1.01, 5.69) | 2.65 (-0.09, 5.4) | 3.27 (-1.43, 7.98) | |
| **Language skills** | Overall | 125 | -1.44 (-3.59, 0.72) | 0.86 (-1.59, 3.31) | | -1.29 (-2.79, 0.22) | | 0.28 (-1.43, 1.99) | 0.96 (-0.49, 2.41) | -1.42 (-3.88, 1.04) | |
|  | Males | 72 | -1.18 (-4.21, 1.87) | 1.25 (-1.75, 4.26) | | -1.03 (-2.93, 0.89) | | 0.7 (-1.59, 3) | 1.48 (-0.46, 3.43) | -0.92 (-4.27, 2.44) | |
|  | Females | 53 | -1.69 (-4.89, 1.51) | 0.2 (-4.17, 4.58) | | -1.68 (-4.22, 0.88) | | -0.16 (-2.87, 2.57) | 0.4 (-1.86, 2.67) | -1.96 (-5.74, 1.83) | |
| **Motor skills** | Overall | 126 | 0.74 (-2.19, 3.66) | 0.14 (-3.18, 3.46) | | -0.42 (-2.49, 1.66) | | -0.85 (-3.18, 1.49) | 0.40 (-1.58, 2.37) | 0.24 (-3.11, 3.59) | |
|  | Males | 74 | -0.56 (-4.9, 3.78) | -1.17 (-5.47, 3.13) | | -1.86 (-4.62, 0.9) | | -2.61 (-5.85, 0.64) | -1.67 (-4.52, 1.18) | -1.93 (-6.74, 2.9) | |
|  | Females | 52 | 1.99 (-1.96, 5.95) | 2.41 (-2.98, 7.81) | | 1.62 (-1.57, 4.81) | | 1 (-2.46, 4.46) | 2.48 (-0.19, 5.17) | 2.5 (-2.16, 7.16) | |
| **Socio-emotional skills** | Overall | 135 | 0.74 (-2.88, 4.37) | 0.40 (-3.60, 4.40) | | 2.33 (-0.18, 4.84) | | 0.82 (-2.01, 3.64) | 0.26 (-2.18, 2.69) | 1.35 (-2.79, 5.48) | |
|  | Males | 82 | -0.27 (-5.03, 4.49) | 0.52 (-4.12, 5.17) | | 2.63 (-0.3, 5.57) | | 1.84 (-1.63, 5.32) | -0.21 (-3.33, 2.92) | 1.09 (-4.14, 6.33) | |
|  | Females | 53 | 1.85 (-3.95, 7.66) | -0.17 (-7.92, 7.59) | | 1.67 (-3.08, 6.42) | | -1.01 (-5.96, 3.95) | 0.63 (-3.4, 4.66) | 1.57 (-5.36, 8.51) | |
| **Adjusted Linear Models for 12 months*** | | | | | | | | | | | |
| **Adaptive skills** | Overall | 130 | -0.64 (-2.81, 1.55) | -1.7 (-4.12, 0.73) | 0.49 (-1.04, 2.02) | | -0.76 (-2.46, 0.95) | | -0.27 (-1.73, 1.19) | | -0.75 (-3.24, 1.76) |
|  | Males | 78 | -0.09 (-2.91, 2.75) | -1.42 (-4.23, 1.41) | 0.89 (-0.92, 2.71) | | -0.72 (-2.8, 1.37) | | -0.95 (-2.81, 0.92) | | -0.22 (-3.33, 2.9) |
|  | Females | 52 | -1.38 (-5.02, 2.27) | -2.11 (-6.91, 2.7) | -0.3 (-3.21, 2.62) | | -0.93 (-4.02, 2.17) | | 0.65 (-1.87, 3.19) | | -1.53 (-5.9, 2.85) |
| **Cognitive skills** | Overall | 127 | -0.64 (-3.37, 2.11) | -0.52 (-3.64, 2.61) | 0.46 (-1.5, 2.44) | | 0.59 (-1.59, 2.79) | | 1.05 (-0.81, 2.91) | | -0.43 (-3.57, 2.71) |
|  | Males | 74 | -3.39 (-7.12, 0.36) | -1.44 (-5.28, 2.41) | -2.05 (-4.51, 0.43) | | -0.77 (-3.69, 2.17) | | -0.56 (-3.1, 1.98) | | -3.78 (-7.93, 0.38) |
|  | Females | 53 | 2.18 (-2.04, 6.42) | 1.29 (-4.43, 7.02) | 4.15 (0.95, 7.35) | | 2.32 (-1.2, 5.85) | | 2.71 (-0.16, 5.59) | | 3.3 (-1.62, 8.23) |
| **Language skills** | Overall | 125 | -1.54 (-3.7, 0.63) | 0.48 (-2.02, 2.99) | -1.37 (-2.91, 0.18) | | 0.14 (-1.6, 1.9) | | 0.95 (-0.51, 2.42) | | -1.54 (-4.02, 0.94) |
|  | Males | 72 | -1.36 (-4.32, 1.61) | 0.37 (-2.67, 3.43) | -0.95 (-2.89, 0.99) | | 0.28 (-2.01, 2.59) | | 1.55 (-0.36, 3.46) | | -1.2 (-4.48, 2.08) |
|  | Females | 53 | -2.04 (-5.24, 1.18) | -0.09 (-4.46, 4.3) | -1.62 (-4.18, 0.95) | | -0.25 (-2.98, 2.5) | | 0.13 (-2.14, 2.42) | | -2.35 (-6.11, 1.43) |
| **Motor skills** | Overall | 126 | 0.7 (-2.16, 3.58) | 0.02 (-3.27, 3.32) | -0.75 (-2.81, 1.33) | | -1.26 (-3.58, 1.06) | | 0.48 (-1.46, 2.43) | | 0.13 (-3.15, 3.43) |
|  | Males | 74 | -0.68 (-4.91, 3.57) | -1.75 (-6.04, 2.55) | -2.31 (-5.04, 0.43) | | -3.23 (-6.39, -0.06) | | -1.27 (-4.07, 1.54) | | -2.06 (-6.74, 2.63) |
|  | Females | 52 | 2.12 (-1.89, 6.14) | 2.42 (-3.01, 7.85) | 1.91 (-1.31, 5.15) | | 1.21 (-2.29, 4.71) | | 2.56 (-0.14, 5.26) | | 2.59 (-2.12, 7.3) |
| **Socio-emotional skills** | Overall | 135 | 0.93 (-2.75, 4.61) | 0.51 (-3.61, 4.65) | 2.63 (0.03, 5.22) | | 0.73 (-2.17, 3.63) | | 0.25 (-2.23, 2.73) | | 1.54 (-2.66, 5.75) |
|  | Males | 82 | -0.25 (-5.1, 4.61) | 0.84 (-4.02, 5.7) | 2.98 (-0.07, 6.05) | | 1.78 (-1.78, 5.36) | | 0.09 (-3.12, 3.31) | | 1.31 (-4.02, 6.65) |
|  | Females | 53 | 2.14 (-3.94, 8.22) | 0.17 (-7.92, 8.27) | 1.73 (-3.28, 6.75) | | -0.94 (-6.16, 4.29) | | 0.67 (-3.51, 4.85) | | 1.79 (-5.42, 9.02) |

**Note:** *Models adjusted for infant sex, gestational age, maternal educational attainment, maternal age, and maternal body mass index category. All sex-specific models adjusted for the same covariates other than infant sex.

**Abbreviations:** β, effect estimate; CI, confidence interval; n, sample size.

**Table S3.** Unadjusted and adjusted linear regression summary of the association between individual placental per- and polyfluoroalkyl substances (PFAS) level (ng/g) and each of the Bayley Scales of Infant and Toddler Development (BSID)-III scores at 24 months in the Glowing cohort, AR, USA, 2010-2014.

|  |  |  | **lnPFOS** | **lnPFNA** | | **lnPFHxS** | | **lnPFDA** | | **lnPFOA** | **ln(sum PFAS)** | |
| --- | --- | --- | --- | --- | --- | --- | --- | --- | --- | --- | --- | --- |
|  |  | **n** | β (95%CI) | β (95%CI) | | β (95%CI) | | β (95%CI) | | β (95%CI) | β (95%CI) | |
| **Unadjusted Linear Models for 24 months** | | | | | | | | | | | | |
| **Adaptive skills** | Overall | 124 | -0.59 (-3.67, 2.49) | -1.98 (-5.47, 1.50) | | 0.53 (-1.64, 2.70) | | 0.21 (-2.29, 2.71) | | -0.97 (-3.05, 1.12) | -0.79 (-4.33, 2.76) | |
|  | Males | 74 | -1 (-5.55, 3.57) | -2.61 (-7.11, 1.89) | | 1.02 (-1.87, 3.92) | | 0.21 (-3.32, 3.76) | | -1.52 (-4.44, 1.41) | -0.77 (-5.8, 4.27) | |
|  | Females | 50 | -0.1 (-4.19, 4.01) | -0.37 (-6.05, 5.33) | | -0.08 (-3.4, 3.26) | | 0.68 (-2.87, 4.25) | | 0 (-2.97, 2.98) | -0.6 (-5.54, 4.34) | |
| **Cognitive skills** | Overall | 125 | -0.45 (-2.85, 1.95) | -0.07 (-2.69, 2.55) | | 0.68 (-0.89, 2.24) | | 0.73 (-1.07, 2.53) | | 0.04 (-1.50, 1.59) | 0.24 (-2.48, 2.97) | |
|  | Males | 74 | -2.32 (-6.42, 1.79) | -0.2 (-3.88, 3.48) | | 1.09 (-1.17, 3.35) | | 1.17 (-1.55, 3.89) | | -0.07 (-2.44, 2.31) | -0.59 (-5.02, 3.84) | |
|  | Females | 51 | 1.05 (-1.48, 3.59) | 0.33 (-3.24, 3.9) | | 0.16 (-1.86, 2.2) | | 0.43 (-1.78, 2.64) | | 0.35 (-1.49, 2.2) | 1.2 (-1.81, 4.22) | |
| **Language skills** | Overall | 118 | 0.10 (-2.74, 2.94) | 0.02 (-3.09, 3.14) | | 1.30 (-0.55, 3.14) | | 1.41 (-0.71, 3.53) | | 0.53 (-1.26, 2.33) | 0.94 (-2.28, 4.15) | |
|  | Males | 67 | -0.25 (-5.34, 4.85) | -0.91 (-5.41, 3.59) | | 1.83 (-0.9, 4.57) | | 0.96 (-2.35, 4.29) | | 0.42 (-2.38, 3.24) | 1.17 (-4.26, 6.6) | |
|  | Females | 51 | 0.4 (-2.52, 3.32) | 1.84 (-2.21, 5.91) | | 0.61 (-1.71, 2.94) | | 2.34 (-0.11, 4.79) | | 0.88 (-1.22, 2.99) | 0.89 (-2.58, 4.37) | |
| **Motor skills** | Overall | 97 | -1.17 (-4.06, 1.71) | -1.40 (-4.53, 1.72) | | 0.73 (-1.13, 2.59) | | 0.88 (-1.30, 3.05) | | -0.27 (-2.09, 1.55) | -0.98 (-4.2, 2.25) | |
|  | Males | 56 | -1.64 (-6.62, 3.34) | -1.07 (-5.13, 3) | | 1.37 (-1.1, 3.85) | | 0.61 (-2.46, 3.7) | | -0.45 (-3.04, 2.16) | -1.02 (-6.09, 4.05) | |
|  | Females | 41 | -0.84 (-4.4,2.73) | -1.96 (-7.2,3.3) | | -0.2 (-3.16,2.76) | | 1.37 (-1.83,4.58) | | 0.11 (-2.58,2.81) | -0.83 (-5.11,3.47) | |
| **Socio-emotional skills** | Overall | 123 | 2.63 (-1.44, 6.70) | 1.29 (-3.38, 5.95) | | 2.71 (-0.12, 5.55) | | 0.30 (-3.06, 3.65) | | 1.61 (-1.17, 4.40) | 3.41 (-1.25, 8.08) | |
|  | Males | 73 | 0.21 (-5.46, 5.9) | 0.14 (-5.54, 5.83) | | 3.48 (-0.03, 6.99) | | 1.3 (-3.18, 5.8) | | 0.7 (-2.99, 4.41) | 1.66 (-4.59, 7.92) | |
|  | Females | 50 | 5.23 (-0.71, 11.18) | 3.93 (-4.5, 12.38) | | 1.62 (-3.34, 6.59) | | -0.55 (-5.87, 4.79) | | 3.01 (-1.36, 7.38) | 5.81 (-1.4, 13.02) | |
| **Adjusted Linear Models for 24 months** | | | | | | | | | | | | |
| **Adaptive skills** | Overall | 124 | -0.55 (-3.64, 2.55) | -1.79 (-5.32, 1.76) | 0.45 (-1.77, 2.68) | | 0.38 (-2.15, 2.91) | | -0.78 (-2.89, 1.34) | | | -0.72 (-4.28, 2.86) |
|  | Males | 74 | -0.93 (-5.43, 3.58) | -2.04 (-6.56, 2.5) | 0.66 (-2.29, 3.62) | | 0.22 (-3.28, 3.73) | | -1.29 (-4.22, 1.65) | | | -0.7 (-5.7, 4.3) |
|  | Females | 50 | -1.04 (-5.24, 3.17) | -1.57 (-7.37, 4.24) | -0.84 (-4.29, 2.63) | | 0.02 (-3.62, 3.67) | | -0.13 (-3.13, 2.88) | | | -1.65 (-6.68, 3.39) |
| **Cognitive skills** | Overall | 125 | -0.2 (-2.6, 2.2) | 0.34 (-2.31, 3) | 0.73 (-0.86, 2.33) | | 0.91 (-0.89, 2.72) | | 0.28 (-1.27, 1.83) | | | 0.6 (-2.12, 3.33) |
|  | Males | 74 | -1.92 (-5.98, 2.16) | 0.63 (-3.1, 4.37) | 1.08 (-1.22, 3.38) | | 1.29 (-1.43, 4.01) | | 0.21 (-2.14, 2.58) | | | -0.07 (-4.47, 4.34) |
|  | Females | 51 | 0.73 (-1.86, 3.33) | -0.13 (-3.75, 3.5) | -0.1 (-2.16, 1.98) | | 0.09 (-2.15, 2.34) | | 0.31 (-1.54, 2.17) | | | 0.87 (-2.19, 3.95) |
| **Language skills** | Overall | 118 | 0.18 (-2.68, 3.04) | 0.03 (-3.14, 3.21) | 1.19 (-0.7, 3.09) | | 1.51 (-0.63, 3.67) | | 0.71 (-1.1, 2.53) | | | 1.04 (-2.2, 4.29) |
|  | Males | 67 | -0.09 (-5.33, 5.17) | -0.67 (-5.38, 4.04) | 1.65 (-1.24, 4.56) | | 1.13 (-2.32, 4.59) | | 0.39 (-2.51, 3.3) | | | 1.24 (-4.34, 6.84) |
|  | Females | 51 | 0.56 (-2.32, 3.45) | 1.88 (-2.09, 5.87) | 0.22 (-2.07, 2.52) | | 2.22 (-0.18, 4.62) | | 1.04 (-0.99, 3.09) | | | 1.06 (-2.34, 4.47) |
| **Motor skills** | Overall | 97 | -1.14 (-4.07, 1.79) | -1.28 (-4.52, 1.97) | 0.65 (-1.27, 2.58) | | 0.77 (-1.48, 3.02) | | -0.11 (-1.97, 1.76) | | | -0.96 (-4.26, 2.35) |
|  | Males | 56 | -1.86 (-6.68, 2.97) | -0.42 (-4.52, 3.7) | 0.91 (-1.62, 3.45) | | 0.31 (-2.72, 3.36) | | -0.45 (-3.02, 2.14) | | | -1.38 (-6.35, 3.6) |
|  | Females | 41 | -0.65 (-4.4, 3.11) | -2 (-7.72, 3.72) | -0.19 (-3.25, 2.88) | | 1.62 (-1.87, 5.13) | | -0.08 (-2.85, 2.7) | | | -0.7 (-5.18, 3.78) |
| **Socio-emotional skills** | Overall | 123 | 3 (-1.1, 7.1) | 1.98 (-2.76, 6.73) | 3.09 (0.18, 6) | | 0.57 (-2.84, 4) | | 1.91 (-0.9, 4.72) | | | 3.89 (-0.81, 8.61) |
|  | Males | 73 | 0.57 (-5.11, 6.26) | 0.88 (-4.89, 6.65) | 3.53 (-0.08, 7.14) | | 1.21 (-3.32, 5.75) | | 0.91 (-2.81, 4.65) | | | 1.93 (-4.34, 8.21) |
|  | Females | 50 | 6.24 (0.03, 12.46) | 5.08 (-3.76, 13.92) | 2.03 (-3.28, 7.35) | | 0.02 (-5.59, 5.64) | | 2.95 (-1.59, 7.5) | | | 6.74 (-0.78, 14.27) |

**Note:** All overall models adjusted for infant sex, gestational age, maternal educational attainment, maternal age, and maternal body mass index category. All sex-specific models adjusted for the same covariates other than infant sex.

**Abbreviations:** β, effect estimate; CI, confidence interval; n, sample size.

**Table S4.** Change in each of the Bayley Scales of Infant and Toddler Development (BSID)-III scores at 12 and 24 months by simultaneously increasing all five placental per- and polyfluoroalkyl substances (PFAS) levels (ng/g) by one quartile, estimated using quantile g-computation, overall and among females and males only in the Glowing cohort, AR, USA, 2010-2014.

|  | **Adjusted Models for 12 months** | | **Adjusted Models for 24 months** | |
| --- | --- | --- | --- | --- |
|  | n | Ψ (95%CI) | n | Ψ (95%CI) |
| **Adaptive Scores** | | | | |
| Overall | 130 | -0.43 (-2.52, 1.66) | 124 | 0.01 (-3.11, 3.14) |
| Males |  | -0.38 (-2.93, 2.17) |  | -0.38 (-4.36, 3.59) |
| Females |  | -1.93 (-5.58, 1.72) |  | 0.13 (-5.18, 5.44) |
| **Cognitive Scores** | | | | |
| Overall | 127 | 0.11 (-2.61, 2.82) | 125 | 0.25 (-2, 2.49) |
| Males |  | -2.55 (-6.03, 0.92) |  | 0.08 (-2.75, 2.92) |
| Females |  | 3.97 (-0.48, 8.42) |  | 0.31 (-3.43, 4.05) |
| **Language Scores** | | | | |
| Overall | 125 | -0.31 (-2.42, 1.81) | 118 | 1.26 (-1.44, 3.96) |
| Males |  | 0.16 (-2.59, 2.91) |  | 0.03 (-3.50, 3.56) |
| Females |  | -0.99 (-4.57, 2.58) |  | 3.25 (-1.19, 7.68) |
| **Motor Scores** | | | | |
| Overall | 126 | -1.35 (-4.29, 1.59) | 97 | 0.83 (-1.88, 3.54) |
| Males |  | -2.86 (-6.63, 0.90) |  | 0.83 (-2.73, 4.39) |
| Females |  | 0.65 (-4.55, 5.85) |  | 0.44 (-4.26, 5.14) |
| **Social-emotional Scores** | | | | |
| Overall | 135 | 2.25 (-1.27, 5.77) | 123 | 3.78 (-0.32, 7.88) |
| Males |  | 1.25 (-3.15, 5.66) |  | 3.26 (-1.92, 8.44) |
| Females |  | 3.82 (-2.54, 10.18) |  | 3.65 (-3.26, 10.55) |

**Note:** All overall models adjusted for infant sex, gestational age, maternal educational attainment, maternal age, and maternal body mass index category. All sex-specific models adjusted for the same covariates other than infant sex.

**Abbreviations:** Ψ, effect estimate; CI, confidence interval; n, sample size.

**Table S5.** Sensitivity analysis for maternal race, breastmilk length, and gestational age for the association between simultaneous exposure to all five placental per- and polyfluoroalkyl substances (PFAS) levels (ng/g) and each of the Bayley Scales of Infant and Toddler Development (BSID)-III scores at 12 and 24 months estimated using quantile g-computation in the Glowing cohort, AR, USA, 2010-2014.

|  | **n** | **Adjusted model** | **Adjusted model with additional adjustment for maternal race** | **Adjusted model with additional adjustment for breastmilk length** | **Adjusted model without adjusting gestational age** |
| --- | --- | --- | --- | --- | --- |
|  |  | Ψ (95%CI) | Ψ (95%CI) | Ψ (95%CI) | Ψ (95%CI) |
| **Adaptive scores** | | | |  |  |
| 12 months | 130 | -0.43 (-2.52, 1.66) | -0.41 (-2.54, 1.71) | -0.6 (-2.89, 1.7) | -0.31 (-2.39, 1.77) |
| 24 months | 124 | 0.01 (-3.11, 3.14) | -0.13 (-3.32, 3.05) | 1.14 (-2.13, 4.42) | -0.09 (-3.21, 3.03) |
| **Cognitive scores** | | | |  |  |
| 12 months | 127 | 0.11 (-2.61, 2.82) | -0.07 (-2.81, 2.66) | 0.77 (-2.13, 3.66) | 0.06 (-2.65, 2.78) |
| 24 months | 125 | 0.25 (-2, 2.49) | -0.27 (-2.49, 1.96) | 0.03 (-2.18, 2.25) | 0.11 (-2.15, 2.37) |
| **Language scores** | | | |  |  |
| 12 months | 125 | -0.31 (-2.42, 1.81) | -0.3 (-2.45, 1.86) | -0.18 (-2.35, 1.99) | -0.26 (-2.38, 1.87) |
| 24 months | 118 | 1.26 (-1.44, 3.96) | 0.65 (-2.04, 3.33) | 1.85 (-0.96, 4.66) | 1.27 (-1.41, 3.95) |
| **Motor scores** | | | |  |  |
| 12 months | 126 | -1.35 (-4.29, 1.59) | -1.3 (-4.28, 1.67) | -1.82 (-4.98, 1.33) | -1.35 (-4.32, 1.61) |
| 24 months | 97 | 0.83 (-1.88, 3.54) | 0.58 (-2.15, 3.31) | 1.24 (-1.66, 4.15) | 0.76 (-1.94, 3.47) |
| **Social emotional scores** | | | |  |  |
| 12 months | 135 | 2.25 (-1.27, 5.77) | 2.7 (-0.83, 6.24) | 2.51 (-1.56, 6.58) | 2.19 (-1.31, 5.69) |
| 24 months | 123 | 3.78 (-0.32, 7.88) | 3.83 (-0.35, 8.02) | 3.8 (-0.52, 8.12) | 3.59 (-0.52, 7.71) |

**Note:** All models at least adjusted for infant sex, gestational age, maternal educational attainment, maternal age, and maternal body mass index category, unless stated otherwise.

**Abbreviations:** Ψ, effect estimate; CI, confidence interval; n, sample size.

**Table S6.** Description of biomatrix-specific levels of per- and polyfluoroalkyl substances (PFAS; ng/g) among those with matched maternal serum (averaged across pregnancy), cord serum and placenta in the Glowing cohort, AR, USA, 2010-2014.

|  |  | **n** | **% above limit of detection** | **Geometric Mean** | **Geometric 95% confidence interval** | **Select Percentiles** | | | | |
| --- | --- | --- | --- | --- | --- | --- | --- | --- | --- | --- |
|  |  |  |  |  |  | **5%** | **25%** | **50%** | **75%** | **95%** |
| **PFOS** | Maternal Serum | 98 | 98 (100%) | 3.17 | 2.85, 3.50 | 1.39 | 2.18 | 3.19 | 4.34 | 7.24 |
|  | Cord Serum | 98 | 96 (98.0%) | 0.97 | 0.83, 1.13 | 0.26 | 0.67 | 1.03 | 1.63 | 2.67 |
|  | Placenta | 98 | 96 (98.0%) | 0.37 | 0.32, 0.43 | 0.11 | 0.244 | 0.406 | 0.593 | 1.01 |
| **PFOA** | Maternal Serum | 98 | 98 (100%) | 0.86 | 0.75, 0.98 | 0.28 | 0.60 | 0.92 | 1.24 | 2.36 |
|  | Cord Serum | 98 | 92 (93.9%) | 0.49 | 0.42, 0.57 | 0.07 | 0.35 | 0.53 | 0.82 | 1.48 |
|  | Placenta | 98 | 72 (73.5%) | 0.05 | 0.04, 0.06 | 0.01 | 0.012 | 0.067 | 0.11 | 0.23 |
| **PFNA** | Maternal Serum | 98 | 98 (100%) | 0.34 | 0.30, 0.39 | 0.10 | 0.22 | 0.37 | 0.54 | 0.90 |
|  | Cord Serum | 98 | 66 (67.3%) | 0.18 | 0.15, 0.21 | 0.07 | 0.07 | 0.2 | 0.32 | 0.57 |
|  | Placenta | 98 | 96 (98.0%) | 0.05 | 0.04, 0.06 | 0.02 | 0.034 | 0.052 | 0.077 | 0.11 |
| **PFHxS** | Maternal Serum | 98 | 98 (100%) | 0.54 | 0.46, 0.65 | 0.14 | 0.28 | 0.54 | 0.91 | 1.91 |
|  | Cord Serum | 98 | 58 (59.2%) | 0.21 | 0.17, 0.27 | 0.07 | 0.07 | 0.19 | 0.48 | 1.89 |
|  | Placenta | 98 | 83 (84.7%) | 0.05 | 0.04, 0.06 | 0.01 | 0.038 | 0.053 | 0.086 | 0.24 |
| **PFDA** | Maternal Serum | 98 | 98 (100%) | 0.15 | 0.13, 0.16 | 0.07 | 0.11 | 0.15 | 0.20 | 0.32 |
|  | Cord Serum | 98 | 22 (22.4%) | 0.08 | 0.08, 0.09 | 0.07 | 0.07 | 0.07 | 0.07 | 0.16 |
|  | Placenta | 98 | 68 (69.4%) | 0.03 | 0.02, 0.03 | 0.01 | 0.007 | 0.036 | 0.052 | 0.07 |
| **summed PFAS** | Maternal Serum | 95 | -- | 1.59 | 4.82, 5.90 | 0.82 | 1.38 | 1.68 | 1.95 | 2.65 |
|  | Cord Serum | 98 | -- | 2.24 | 2.00, 2.50 | 0.91 | 1.66 | 2.19 | 3.08 | 5.57 |
|  | Placenta | 98 | -- | 0.6 | 0.52, 0.68 | 0.22 | 0.389 | 0.625 | 0.903 | 1.71 |

**Note:** Maternal and cord serum levels are measured in ng/mL whereas placental levels are measured in ng/g. Maternal serum levels are average across trimesters (that is, PFAS levels collected in trimesters 1, 2 and 3).

**Abbreviations:** n, sample size.
